# Structurally optimized analogs of the retrograde trafficking inhibitor Retro-2cycl limit *Leishmania* infections

**DOI:** 10.1371/journal.pntd.0005556

**Published:** 2017-05-15

**Authors:** Evan Craig, Charles-Eugene Huyghues-Despointes, Chun Yu, Emma L. Handy, Jason K. Sello, Peter E. Kima

**Affiliations:** 1 Department of Microbiology and Cell Science, University of Florida, Gainesville, Florida, United States of America; 2 Department of Chemistry, Brown University, Providence Rhode Island, United States of America; Ohio State University, UNITED STATES

## Abstract

In infected mammalian cells, *Leishmania* parasites reside within specialized compartments called parasitophorous vacuoles (LPVs). We have previously shown that Retro-2, a member of a novel class of small retrograde pathway inhibitors caused reduced LPV sizes and lower parasite numbers during experimental *L*. *mexicana* sp. infections. The purpose of this study was to determine if structural analogs of Retro-2^cycl^ reported to have superior potency in the inhibition of retrograde pathway-dependent phenomena (*i*.*e*., polyomavirus cellular infection by polyomavrius and Shiga toxin trafficking in cells) are also more effective than the parent compound at controlling *Leishmania* infections. In addition to their effects on LPV development, we show that two optimized analogs of Retro-2^cycl^, DHQZ 36 and DHQZ 36.1 limit *Leishmania amazonensis* infection in macrophages at EC_50_ of 13.63+/-2.58μM and10.57+/-2.66μM, respectively, which is significantly lower than 40.15μM the EC_50_ of Retro-2^cycl^. In addition, these analogs caused a reversal in *Leishmania* induced suppression of IL-6 release by infected cells after LPS activation. Moreover, we show that in contrast to Retro-2^cycl^ that is *Leishmania* static, the analogs can kill *Leishmania* parasites in axenic cultures, which is a desirable attribute for any drug to treat *Leishmania* infections. Together, these studies validate and extend the published structure-activity relationship analyses of Retro-2^cycl^.

## Author summary

Over 12 million people worldwide are infected by *Leishmania* parasites and many more are at risk of being infected. In the mammalian host, *Leishmania* parasites live in intracellular compartments called parasitophorous vacuoles (LPVs). We have previously shown that Retro-2, a member of a novel class of small retrograde pathway inhibitors, caused reduced LPV sizes and lower parasite numbers during *L*. *mexicana* sp. infections. The purpose of this study was to determine if analogs of Retro-2^cycl^ reported to have superior potency in the inhibition of retrograde pathway-dependent phenomena are also more effective than the parent compound in controlling *Leishmania* infections. We show that two optimized analogs of Retro-2^cycl^ are significantly more effective than the parent compound at controlling *Leishmania* infections and in the reversal of parasite induced suppression of IL-6 release by infected cells after LPS activation. Moreover, we show that the analogs kill *Leishmania* parasites in axenic cultures, which is a desirable characteristic for any compound that is being considered to treat *Leishmania* infections. Together, these studies validate and extend the published structure-activity relationship analyses of Retro-2^cycl^. In addition, they show that the Retro-2^cycl^ analogs reverse the effects of the parasite on macrophage responses that are likely to affect the overall host response to infection.

## Introduction

Leishmaniasis is a disease with clinical presentations that range from cutaneous lesions to visceral disease. Several *Leishmania* species including parasites in the *Leishmania mexicana* complex (*L*. *mexicana*, *L*. *amazonensis*, *L*.*pifanoi*) are the causative agents of cutaneous leishmaniasis. Visceral leishmaniasis is most often caused by *L*. *donovani* and *L*. *infantum*. In the mammalian host, *Leishmania* parasites live in intracellular compartments called parasitophorous vacuoles (LPVs). There is ample evidence that during the biogenesis and maturation of LPVs, they acquire characteristics of late endocytic pathway compartments such as acidification and they display numerous molecules that otherwise are resident in the late endoyctic pathway including the Lysosome associated membrane protein (LAMP1) and cathepsins [1]. That notwithstanding, there is still much that remains to be learned about the composition of LPVs. A few studies have shown that some molecules that are otherwise localized to or associated with secretory pathway compartments are displayed on LPVs as well [2][3]. These molecules include the endoplasmic reticulum resident, calnexin and several soluble N-ethylmaleimide-sensitive fusion attachment protein receptors (SNAREs) such as syntaxin 5 (Stx5), 18 and sec22b that mediate the fusion of early secretory pathway vesicles [4]. These SNARE molecules were shown to be functionally relevant to LPV biogenesis with evidence that expression of dominant negative variants or when their protein levels in cells were knocked down, the result was that smaller LPVs developed that harbored much fewer *L*. *mexicana* complex (*L*. *pifanoi* and *L*. *amazonensis*) parasites [3]. These observations imply that pharmacological intervention in protein transport could be a viable strategy for the treatment of *Leishmania*.

Recently, it was reported that a small molecule called Retro-2^cycl^ and characterized by a dihydroquinazolin-4-one (DHQZ) core structure disrupts the retrograde transport machinery in mammalian cells [5]. Retro-2^cycl^ was then shown to block processes that depend on retrograde transport, including ricin and Shiga toxin engorgement of mammalian cells. After their lectin receptor mediated entry into cells, ricin and Shiga toxin are transported through the retrograde route to the cell cytosol where they block protein synthesis [6]. Retro-2^cycl^ has also been shown to block the entry into cells of some DNA viruses including polyomaviruses and papillomaviruses that are known to be internalized via the retrograde pathway [7]. In two separate Structure Activity Relationship (SAR) studies that followed, analogs of Retro-2^cycl^ were described that can more potently block ricin and Shiga toxin trafficking in cells [8] and also block polyomavirus and papillomavirus infection of cells [7]. Interestingly, while both studies reached many of the same conclusions, their most potent inhibitors of the retrograde trafficking had subtly different and unique structures. Among the differences was that the most potent compound in the Shiga toxin study had a fused thiophene-thiazole moiety appended to C2 of the dihdroquinazolinone structure whereas that in the viral infection study (DHQZ 36) had an ethyl-thiophene at the same position. Further, the most active compound in the latter study had a 4-fluorbenzyl substituent at N3 while the former had a phenyl group at the same position. Because each study indicated that the aforementioned substituents were both highly associated with inhibitor potency, we prepared a hybrid compound having a fused thiophene-thiazole at C2 and a 4-fluorobenzyl substituent at N3 called DHQZ 36.1. We anticipated that it would be markedly more potent that both Retro-2^cycl^ and DHQZ 36 as an inhibitor of retrograde trafficking.

Retro-2 was previously shown to limit macrophage infections by parasites of the *L*. *mexicana* complex [3]. That study showed that treatment of infected macrophages with Retro-2 blocked the development of LPVs. Specifically, LPVs in the treated macrophages remained tight with minimal vacuolar space rather than distending to sizes that are greater than the infected cell nucleus. Moreover, Retro-2^cycl^ treated macrophages had LPVs that contained significantly fewer parasites than LPVs in the negative controls. This latter observation suggested that Retro-2 might also have a direct effect on parasite viability. In this study, we present results from an evaluation of Retro-2^cycl^ and two optimized analogs (DHQZ 36 and DHQZ 36.1) on axenic cultures of *Leishmania* promastigotes and also parasites in macrophage infections. We found that Retro-2^cycl^ is *Leishmania* static, whereas the analogs are *Leishmania*-cidal. We also discovered that treatment of infected macrophages with the retrograde trafficking inhibitors could reverse *Leishmania* induced suppression of IL-6 release after LPS activation. In some assays, DHQZ 36.1 was found to be more efficacious than DHQZ 36, which validates the SAR studies and points the way towards the development of retrograde trafficking inhibitors for the treatment of infectious diseases.

## Materials and methods

### Chemicals and drugs

Retro-2^cycl^, DHQZ 36 and DHQZ 36.1 were synthesized by Dr. Jason Sello at Brown University as described previously (7) and in Supplement 1. Stock solutions were prepared in dimethylsufloxide (DMSO). Retro-2^cycl^ was also purchased from Sigma-Aldrich (CAS: 1429192-00-6) and prepared in DMSO as well. Miltefosine was purchased from Sigma-Aldridge (Catalog# m5571) and a stock solution was prepared in NanoPure diH20. The tetrazolium salt MTT [3-(4, 5-dimethylthiazol-2-yl)-2,5-diphenyl tetrazolium bromide] Cell Viability Assay Kit was purchased from Biotium (Fremont, CA). L-azidohomoalanine (AHA), PEG4 carboxamide-propargyl biotin (biotin alkyne) and the Click-iT reaction buffer kit were purchased from ThermoFisher Scientific (Waltham MA). AHA and biotin alkyne stock solutions were prepared in DMSO at 50mM and 400mM, respectively.

### Cell cultures

Promastigotes of *L*. *amazonensis* strain RAT/BA/74/LV78 (LV78) obtained from Dr. Lynn Soong (UTMB, TX) were maintained at room temperature in Schneider’s Drosophila medium supplemented with 10% heat-inactivated fetal bovine serum (FBS) and 10μg/mL Gentamycin. Promastigotes of *L*. *donovani* (MHOM/SD/62/1S-C1_2D_ (SD)) obtained from Dr. Hira L. Nakhasi laboratory (FDA, MD) were maintained at 26°C in M199 medium supplemented with 10% FBS and 1% penicillin/streptomycin. RAW264.7murine macrophages were obtained from ATCC and maintained in Dulbecco’s Modified Eagle’s Medium (DMEM) supplemented with 10% FBS and 1% streptomycin-penicillin at 37°C with 5% CO_2_.

### Promastigote drug susceptibility assay

To determine the EC_50_ of Retro-2^cycl^ and its analogs on axenic parasites, an MTT Cell Viability Assay Kit was used. Early stationary phase promastigotes were seeded at 1x10^5^ parasites/well in a 96-well tissue culture plate and allowed to grow for 48 hrs at room temperature in the presence of Retro-2^cycl^, DHQZ 36 or DHQZ 36.1 with concentrations ranging from 0–100μM for *L*. *amazonensis* treatments and 0–200μM for *L*. *donovani* treatments. Miltefosine treatments of axenic promastigotes were in concentrations ranging from 0–100μM. Parasite susceptibility to the DMSO vehicle alone was assessed by treating parasites at an equal concentration of DMSO to the highest concentration of drug used in each experiment. Promastigotes were incubated with MTT for 2 hrs and formazan product was read at 570 nm wavelength and a 630 nm background wavelength as described by the Biotium protocol. Viable parasites were estimated from an MTT standard curve that was made from serial dilutions of parasites and correlation of those values to the relative amount of formazan product. Plots of % Cell Viability as compared to controls vs. Log Molar Concentration were generated in GraphPad Prism 7. EC_50_s were calculated by nonlinear regression analysis of the sigmoidal curves that were generated. Significance of the differences in parasite growth was determined using the multiple t-tests function. Statistical significance between time points of each concentration was measured using a two-way ANOVA in GraphPad Prism 7 with the Holm-Sidak posthoc test for multiple comparisons

### Promastigote recovery

To determine if the inhibitory effect of Retro-2^cycl^ and its SAR analogs on promastigote parasites was transient, a recovery experiment was performed on promastigotes. Promastigotes were seeded at 1x10^5^ parasites per well and treated with concentrations of Retro-2^cycl^, DHQZ 36, DHQZ 36.1 and miltefosine ranging from 0–100μM for 72 hrs. After treatment, parasites were collected and spun down. Drugs were washed from parasites with fresh parasite medium and resuspended in 100μL fresh parasite medium. 50μL of the parasites were used for MTT assay for the 72 hrs time point and the other 50μL was plated in a new 96-well plate with fresh parasite medium. These cells were allowed to grow for 48 hrs before performing the MTT assay. Cell numbers were estimated by comparison of formazan product to a standard generated at time 0 with known numbers of parasites. Formazan products at 48 hrs after recovery was then compared to amounts produced at 72 hrs of treatment to determine if growth occurred. The experiment was repeated at least three times with three replicates per concentration. Graphs were generated in GraphPad Prism 7 and significance of differences in growth was determined using a using a two-way ANOVA with the Holm-Sidak posthoc test for multiple comparisons.

### Drug treatment of infected RAW264.7murine macrophages

These experiments were performed as described previously (3). Briefly, macrophages were plated in 100 mm Petri dishes containing sterile glass coverslips and allowed to adhere overnight in complete DMEM media at 37°C with 5% CO_2_. Macrophages were infected with mid-stationary phase promastigotes at 1:10 or 1:20 ratio for 24 hrs. Coverslips were then washed and treated with the varying concentrations of Retro-2^cycl^, DHQZ 36, DHQZ 36.1 and miltefosine as described in the experiments. Some coverslips were incubated in DMSO as vehicle control. After incubation with drugs for an additional 24 hrs at 34°C, coverslips were fixed in methanol for 5 minutes prior to Giemsa staining. After methanol fixation, infected cells were stained with Wright-Giemsa stain at a 1:20 dilution for 15 minutes made fresh with NanoPure diH_2_O. Cells were then washed twice with NanoPure diH_2_O and allowed to dry for 1–2 hrs. Coverslips were dehydrated with xylenes for 1 minute and mounted in Permount on glass slides for viewing under a bright field light microscope. The percentage of infected macrophages and the average number of parasites was determined by counting at least 200 macrophages per coverslip. Counts were done in duplicate over at least three experiments and EC_50_ values were determined in GraphPad Prism using a three parameter dose-response best-fit curve line. Statistical significance between treated infected cells and the control was measured using a one-way ANOVA in GraphPad Prism 7.

### Determination of vacuole size

The protocol for determining vacuole size was as described previously [3]. Briefly, RAW264.7macrophages on coverslips were infected with stationary *L*. *amazonensis* promastigotes at 1:20 ratio (macrophage to parasites) for 4 hrs after which parasites were washed off and DHQZ analogs or miltefosine were added. Infected macrophages were fixed with 2% PFA then processed in immunofluorescence assays to visualize the distribution of LAMP1. IFAs were visualized and captured using a QImaging Retiga 1300C cooled CCD camera mounted on an Olympus BX50 microscope equipped with automated filters with 100x NA 1.30 oil-immersion objective. Scored LPVs were delimited by LAMP-1 reactivity and contained at least one parasite nuclei visualized by DAPI stain. Vacuole size was determined by measuring vacuole area via ImageJ. At least 30 vacuoles were measured per concentration per experiment. Statistical significance between treated infected cells and the control was measured using a one-way ANOVA in GraphPad Prism 7 with the Dunnett test for multiple comparisons

### Cytokine secretion of RAW264.7 macrophages during infection

Macrophages were plated in 6-well plates at a density of 5x10^5^ cells/mL overnight for adherence. The cells were then infected at a 1:20 ratio with stationary *L*. *amazonensis* promastigotes as described above. After 24 hrs infected wells were treated with Retro-2^cycl^, DHQZ 36, DHQZ 36.1 at 12.5, 50 or 100μM or miltefosine at 1, 5 or 10μM for 24 hrs in complete DMEM supplemented with 100ng/ml or 500ng/mL LPS and 100ng/mL IFN-γ. Controls were treated with complete DMEM alone or LPS/IFN-γ without drugs or with drugs without LPS/IFN-γ stimulation. Media was collected from plates after 24 hrs of treatment and spun down to remove any cell debris. Supernatants fluids were then assessed in ELISA as described previously [3].

### Statistical analysis of results

Data analysis and the generation of graphs were performed using Microsoft Excel and GraphPad Prism 7 (La Jolla, CA) as described above. Data are presented as the mean ± standard deviation. Statistical significance between time points of each concentration and also between treated infected and uninfected cells was measured using a one-way or two-way ANOVA (dependent on the experimental details) in GraphPad Prism 7. This was followed in most situations by a post hoc test that considers multiple comparisons. Specifically, the Holm-Sidak or the Dunnett post hoc tests were used.

## Results

### Retro-2^cycl^ and analogs thereof suppress *Leishmania* infections of macrophages

**Fig 1:**
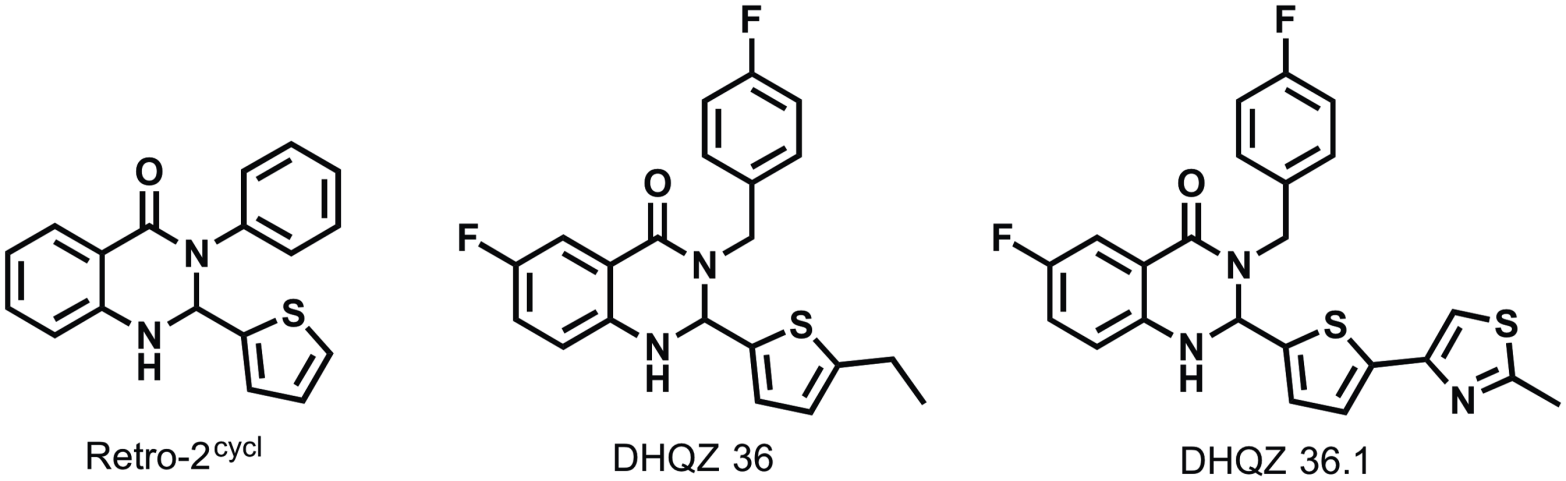
Structures of retrograde trafficking inhibitors. Retro-2^cycl^ is the bioactive isomer of Retro-2 reported in (5). DHQZ 36 is a more biologically active analog of Retro-2^cycl^ reported by Carney et al [7]. DHQZ 36.1 is an analog of DHQZ 36 having the fused thiophene-thiazole moiety at C2 of the dihydroquinazolin-4-one.

Consistent with predictions that trafficking of vesicles was important for *Leishmania* infections, we reported that that the retrograde trafficking inhibitor Retro-2 suppressed infection of macrophages by *Leishmania* [3]. A subsequent report stating that Retro-2 was a mixture of the initially disclosed acyclic isomer along with a cyclic isomer Retro-2^cycl^ and that the cyclic form was biologically active motivated a review of the compound’s activity [9][10]. Indeed, SAR studies of Retro-2^cycl^ confirmed its biological activity and yielded analogs with markedly enhanced potencies as inhibitors in ricin and Shiga toxin cell engorgements assays [8] and cell infectivity assays with polyoma- and papillomaviruses [7]. Though the two SAR studies of Retro-2^cycl^ were largely consistent, there were some key differences. For instance, the Noel et al [8] study found that compounds with a methyl substituent on N1 of the dihydroquinazolin-4-one exhibited improved activity in the Shiga toxin assays whereas Carney et al [7] found that methyl substitution at this position were less active in the viral infectivity assays. It is not clear whether this discrepancy reflects differences in the assay and/ cell line that is used. Aside from the aforementioned methyl substituent, the Retro-2^cycl^ analogs differ with respect to the substituents at C2 and N3 of the dihydroquinazolin-4-one; which are a methyl thiophene and phenyl groups in Retro-2, respectively. In the study by Noel et al [8], it was reported that the most potent DHQZ had a fused thiophene-thiazole moiety at C2 and a phenyl group at N3. In contrast, the most efficacious compound in the Carney et al experiments [7] called DHQZ 36 had a 2-ethyl thiophene at C2 and a 4-fluorobenzyl group at N3 (Fig 1). Based on both SAR studies, we predicted that an analog of DHQZ 36 having the fused thiophene-thiazole moiety at C2 (Retro-2.1a (2S)) also described in the Noel study would be particularly active. We synthesized and named this compound DHQZ 36.1 (Fig 1). The synthesis scheme and properties of the DHQZ compounds used in this study are shown in supplemental figures (S1 Text; S1, S2 and S3 Figs).

**Fig 2:**
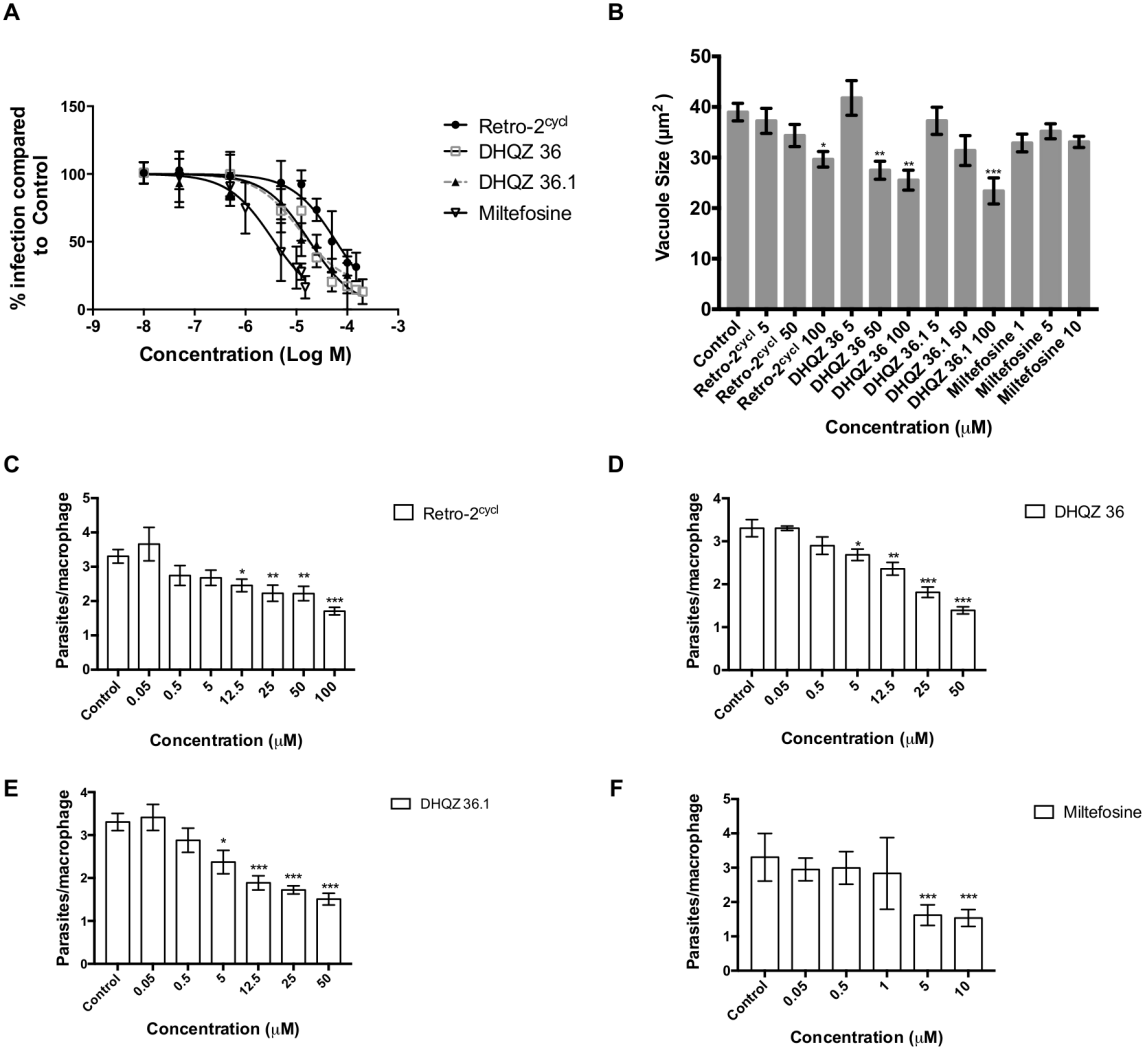
DHQZ SAR derivatives limit *L*. *amazonensis* infections of RAW264.7 macrophages. Macrophages were infected for 24 hrs and then treated with the indicated compounds. Coverslips were then fixed with methanol and stained with Wright Geimsa stain. At least 200 macrophages were counted by light microscopy to determine drug efficacy. (A). The number of infected cells at the indicated drug concentrations was determined. Infection values were standardized relative to control and plotted against the drug concentration (M = Molar) in GraphPad Prism 7. B). For determination of vacuole sizes, 4 hrs old infection were incubated with DHQZ analogs or miltefosine at concentrations ranging from 0–100 μM and 0–10 μM, respectively and incubated for an additional 44 hrs. LPVs were visualized after performing immunofluorescence assays using a rat anti-murine LAMP-1 antibody and parasite nuclei were visualized using DAPI. Vacuole areas were measured in ImageJ from at least 30 vacuoles per concentration in each experiment. Bars are representative of triplicate experiments. C-F). Plots of parasite burden as determined by the number of parasites per infected RAW264.7 macrophages after treatment with DHQZ analogs and miltefosine treatment is shown. Statistical significance was determined by one-way ANOVA in GraphPad Prism 7 with the Dunnett posthoc test for multiple comparisons (* = p-value < 0.05, ** = p-value < 0.01, *** p-value < 0.001).

To test our predictions about the high potency of DHQZ 36.1, we elected to compare its activity to that of Retro-2^cycl^ and DHQZ 36 in suppressing the infection of macrophages by *Leishmania*. We had reported previously that Retro-2 limits growth of *Leishmania* parasites not only in infected macrophages but also in infected animals [3]. In those studies, Retro-2^cycl^ had no discernable toxicity to macrophages even after incubation with upwards of 200μM of Retro-2^cycl^. Here, the efficacies of Retro-2^cycl^, DHQZ 36, and DHQZ 36.1 in limiting parasite infection in macrophages were assessed. Our focus was *L*. *amazonensis*-infected cells. RAW264.7macrophage infections were established for 24hrs after which the drugs were added and the cultures were scored after an additional 48hrs by enumeration of infected cells stained with Wright-Geimsa. Reduction in the number of infected cells implied that incubation with the drugs resulted in clearance of the amastigote forms within macrophages. All three compounds reduced the number of infected cells. The estimated EC_50_ of each compound that was derived from the curves in Fig 2(A) are 40.15+/-4.46μM for Retro-2^cycl^, 13.63+/-2.66μM for DHQZ 36 and 10.57+/2.58μM for DHQZ 36.1. Although the analogs were markedly more potent than Retro-2^cycl^, we found that DHQZ 36.1 exhibited slightly better efficacy than DHQZ 36 in this assay. In parallel studies, we evaluated the efficacy of miltefosine and found that its EC_50_ was 2.84+/-0.79μM. Retro-2^cycl^ and its analogs exhibit low toxicity to macrophages. An LDH assay that assessed the toxicity of Retro-2^cycl^, DHQZ 36 and DHQZ 36.1 to RAW264.7macrophages showed that the effect of these drugs on macrophages was very limited (S4 Fig; S4 Text). It is noteworthy that previous studies had found Retro-2 has very low toxicity, if any, in animal studies [3][5]. Together, these observations suggest that Retro-2^cycl^ and its SAR analogs have a high therapeutic index. Miltefosine is an anti-*Leishmania* drug that is currently in use. The cytotoxicity of miltefosine to mouse macrophages has been estimated at an EC_50_ of ~26μM [11].

Retro-2^cycl^ had been shown to reduce the size of *Leishmania* PVs in macrophages by blocking Stx5 mediated fusion of secretory pathway vesicles to PVs [3]. We therefore evaluated LPV sizes in the presence of the DHQZ analogs. Fig 2B shows that with increasing concentrations of DHQZ 36 and DHQZ 36.1 there is a significant diminution in the size of LPVs. DHQZ 36 treatment had the most striking effect; vacuole sizes were reduced by 30% at 50μM. DHQZ 36.1 reduced vacuole size at 50 and 100μM by 25% and 40%, respectively. These parasites also caused a reduction in the number of parasites per macrophage. Significant parasite loss was observed with as low as 5μM after treatment with DHQZ 36 and DHQZ 36.1 (Fig 2C, 2D, 2E and 2F). As expected, miltefosine treatment resulted in clearance of parasites but it did not lead to any significant effect on vacuole size even at 10μM (Fig 2B). These drug induced reductions in the intracellular parasite burden wasn’t as a result of nitric oxide induction (results of NO measurements in S5 Fig; S5 Text)

### Retrograde trafficking inhibitors are active against *Leishmania* promastigotes

**Fig 3:**
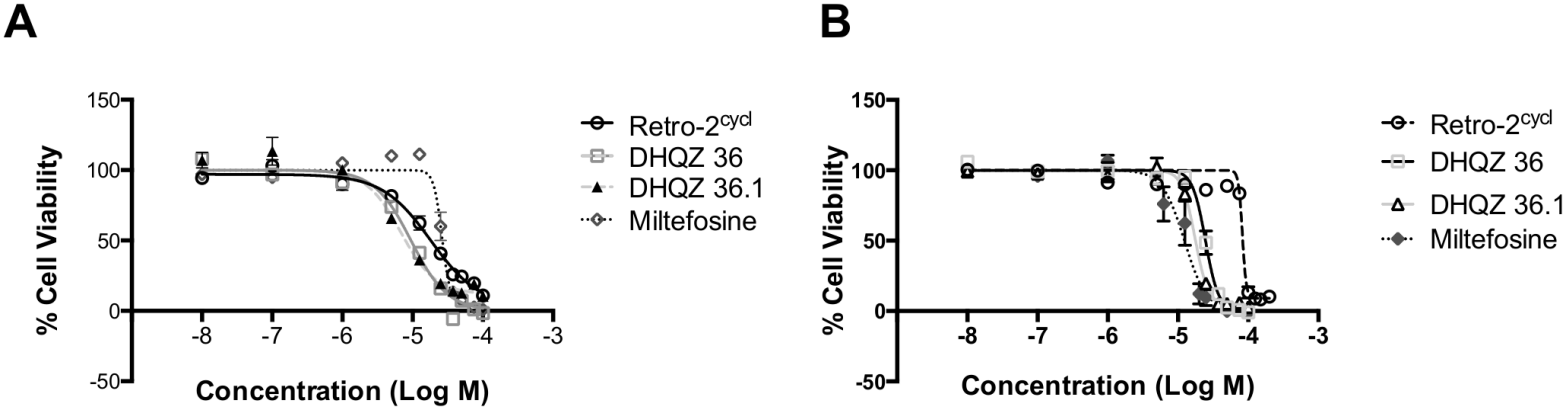
DHQZ SAR analogs are more potent than Retro-2^cycl^ on *Leishmania* promastigotes. *Leishmania* promastigotes were incubated with increasing concentrations of Retro-2^cycl^ or the DHQZ SAR analogs for 48 hours. For comparison, parasites were incubated in miltefosine as well. The viability of *L*. *amazonensis* (A) or *L*. *donovani* (B) parasites after drug treatment was determined in MTT assays. Estimated parasites after drug treatment were expressed relative to control parasites. The data was entered into GraphPad Prism 7 from which the sigmoidal curves were generated. Experiments were run at least three times and curves were analyzed by non-linear regression to obtain EC_50_ values.

Because DHQZ 36 and DHQZ 36.1 reduced both the size of the *Leishmania* PVs and the number of parasites they contained, we investigated the possibility that the compounds were also directly toxic to the pathogen. Initially, the effects of these compounds on axenic *L*. *amazonensis* promastigotes were assessed in MTT cell viability assays. Parasites were incubated in the presence of increasing concentrations of Retro-2^cycl^, DHQZ 36 and DHQZ 36.1. The compounds were evaluated in parallel with miltefosine. Fig 3A shows dose response effects of these compounds on *L*. *amazonensis* after incubation for 48hrs. The data was analyzed by GraphPad Prism 6 from which 4-parameter curves were generated. From these curves the EC_50_ of each drug was estimated at 18.2+/-3.96 μM, 9.83+/-1.04 μM and 6.12+/-0.34 μM for Retro-2^cycl^, DHQZ 36 and DHQZ 36.1, respectively (Table 1). The EC_50_ for miltefosine on *L*. *amazonensis* in these experiments was estimated at 27.13+/-3.2μM. These observations suggest that DHQZ 36 and DHQZ 36.1 are more effective at killing *L*. *amazonensis* parasites than Retro-2^cycl^ and miltefosine. We proceeded to also evaluate the effects of these compounds on *L*. *donovani* promastigotes. The effects of these compounds on *L*. *donovani* are shown in Fig 3B. The EC_50_ for each drug that was calculated from those curves were 84.3+/-4.6 μM, 24.7+/-4.6 μM and 17.6+/-2.3 μM for Retro-2^cycl^, DHQZ 36 and DHQZ 36.1 respectively (Table 1). The EC_50_ of miltefosine on *L*. *donovani* promastigotes was 12.34+/-2.22 μM. In contrast to their efficacy on *L*. *amazonensis*, the DHQZ compounds are effective at higher concentrations on *L*. *donovani* parasites. The reverse is true for miltefosine. As the DHQZs are non-toxic to mammalian cells at the aforementioned concentrations, one could conclude that the essential retrograde trafficking pathway is more sensitive in *Leishmania*. Nevertheless, as was the case in the viral infectivity assays [7], DHQZ 36 and DHQZ 36.1 are more efficacious than Retro-2^cycl^ in suppression of *Leishmania* infections. These results corroborate the SAR studies and the proposed mode of action of the compounds. The SAR of these retrograde trafficking inhibitors was advanced by our observations that in some assays DHQZ 36.1 is more potent that DHQZ 36.

**Table 1:** EC_50_ of Retro-2cycl and DHQZ SAR analogs on *Leishmania* promastigotes.

EC_50_ (μM)		
Treatment	*L*. *amazonensis*	*L*. *donovani*
Retro-2^cycl^	18.45 ± 3.96	84.3 ± 4.60
DHQZ 36	9.83 ± 1.04	24.70 ± 4.60
DHQZ 36.1	6.12 ± 0.34	17.62 ± 2.3
Miltefosine	26.22 ± 3.86	12.34 ± 2.22

The EC_50_ of each drug was calculated from the curves in Fig 3A and 3B. EC_50_ of DHQZ 36 and DHQZ 36.1 on *L*. *amazonensis* or *L*. *donovani* are significantly more effective than Retro-2^cycl^ (p-value <0.001). On *L*. *amazonensis* DHQZ 36.1 improves on DHQZ 36 (p-value <0.001. On *L*. *donovani*–DHQZ 36.1 also improves on DHQZ 36 (p-value <0.001)).

### Optimized analogs of Retro-2 are *Leishmania*-cidal

**Fig 4:**
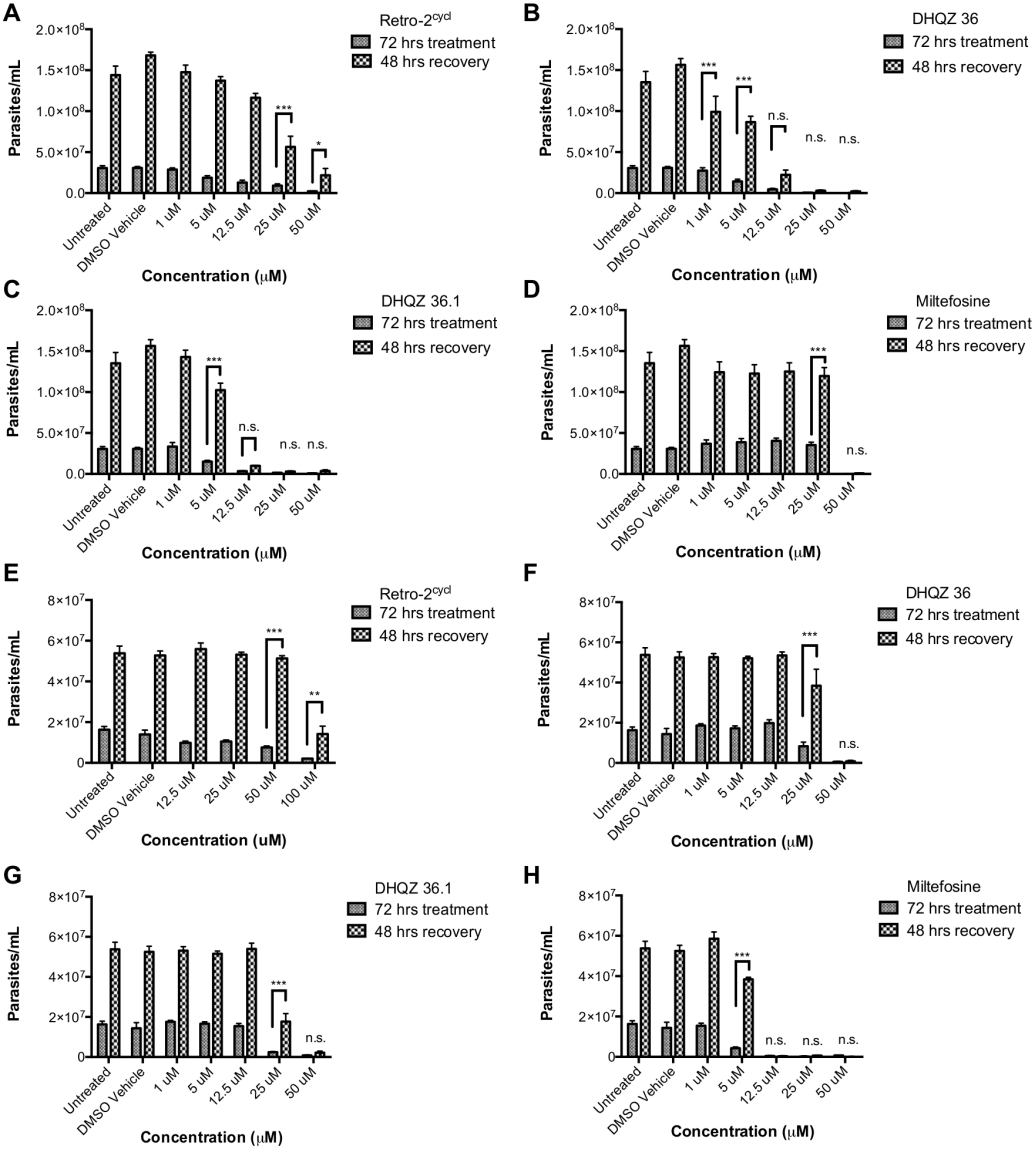
DHQZ SAR analogs cause death of *Leishmania* parasites. *Leishmania* promastigotes were seeded at 1x10^5^ cells/mL and incubated with increasing concentrations of each test compound. After 72 hrs the parasites were washed to remove the drugs. The number of parasites in half of the sample was were then estimated in MTT assays. The other half was resuspended in fresh media and incubated for an additional 48hrs to determine if parasites could recover from drug treatment. The number of parasite were then estimated in MTT assays. The estimate of viable parasites after drug treatment and after recovery are presented. (A–D) show results of each drug on *L*. *amazonensis* parasites. (E–H) show results of each drug on *L*. *donovani* parasites. The results were compiled from at least 3 replicate experiments and were evaluated for statistical significance using a using a two-way ANOVA in GraphPad Prism 7 with the Holm-Sidak posthoc test for multiple comparisons (* = p-value < 0.05, ** = p-value < 0.01, *** p-value < 0.001).

In previous studies with Retro-2, we had found that it inhibited the replication of the parasites [3]. In the MTT assays described above, it appeared that the DHQZs could kill the *Leishmania* concentrations in the micromolar (μM) range. For an in-depth analysis of the extent of killing, the parasites were treated with Retro-2^cycl^ and the analogs thereof for 72 hrs, washed, and then cultured in fresh media lacking the compound. Growth recovery of parasites was ascertained via the MTT assay after an additional 48 hrs of culture. Interestingly, we found that *L*. *amazonensis* parasites were able to resume growth after treatment with Retro-2^cycl^ at concentrations as high as 50μM (Fig 4A). Although the EC_50_ of Retro-2^cycl^ was estimated to be less than 25μM, parasites recovered after incubation in much higher drug concentrations, which implies that the drug caused reduced parasite metabolism but not parasite death. In contrast, parasites were unable to recover growth when treated with DHQZ 36 and DHQZ 36.1 at concentrations at or above 12.5μM (Fig 4B and 4C). Reflecting its greater potency in the aforementioned assay, we found that fewer parasites were able to recover from treatment with 12.5μM of DHQZ 36.1 than from treatment with DHQZ 36 at the same concentration (Fig 4B and 4C). We also found that only a proportion of the initial inoculum of parasites was observed after incubation with DHQZ 36 and DHQZ 36.1, which suggested that these compounds caused parasite death and clearance. The effect of miltefosine on parasites was evaluated for comparisons. There was robust *L*. *amazonensis* parasite recovery after incubation in 25μM miltefosine (Fig 4D), which is consistent with the EC_50_ of miltefosine on *L*. *amazonensis*. However, no parasites recovered after incubation in 50μM miltefosine.

Analogous recovery experiments were performed with *L*. *donovani* parasites. *L*. *donovani* recovered from incubation with up to 100μM Retro-2^cycl^ (Fig 4E). Parasite growth recovery from incubation with 50μM of DHQZ 36 and DHQZ 36.1 was very limited if at all; however, there was robust growth recovery from incubation with 25μM of either DHQZ 36 or DHQZ 36.1 (Fig 4F and 4G). In contrast, *L*. *donovani* parasites did not recover from incubation with miltefosine at 12.5μM or higher concentrations (Fig 4H). The observation that miltefosine exerts a more potent effect on *L*. *donovani* as compared to *L*. *amazonensis* is consistent with reports by others that miltefosine is most effective on *L*. *donovani* parasites and that it exhibits variable efficacy on other *Leishmania* species [12]. Taken together, these studies show that the optimized analogs of Retro-2^cycl^ share the *Leishmania*-cidal properties with miltefosine, whereas the less potent Retro-2^cycl^ is *Leishmania*-static.

### DHQZ compounds reverse inhibition of cytokine production by infected cells

Several studies have shown that macrophages infected with *Leishmania* parasites are defective in their release of pro-inflammatory cytokines in response to stimulation by lipopolysaccharide (LPS) with or without IFNγ [13],[14],[15]. Although there isn’t a consensus on the mechanism by which inhibition of cytokine release by infected cells is achieved, some of the proposed mechanisms have implicated parasite molecules that are released from LPVs that then interfere with signal transduction intermediates [13][16]. Retro-2^cycl^ is known to function by disrupting Stx5 that exerts its functions in the secretory pathway. Although we found no evidence that it blocks secretion of macrophage proteins from non-infected cells [3], it is possible that it could block protein secretion in *Leishmania* parasites. To assess whether DHQZ 36 and DHQZ 36.1 can block secretion from axenic parasites, the secretion of metabolically labeled parasite proteins was evaluated. Parasites were metabolically labeled with L-azidohomoalanine (AHA) in a Click-iT protocol ([17] and S6 Text). Parasite secretion was then monitored by sampling the culture medium of parasites that were grown in the presence of Retro-2^cycl^, DHQZ 36 or DHQZ 36.1. Both DHQZ 36 and DHQZ 36.1 caused over 40% reduction in secreted of parasite proteins (S6 Fig; S6 Text). It is therefore likely that these compounds cause a reduction in parasite protein secretion from parasites within infected cells.

**Fig 5:**
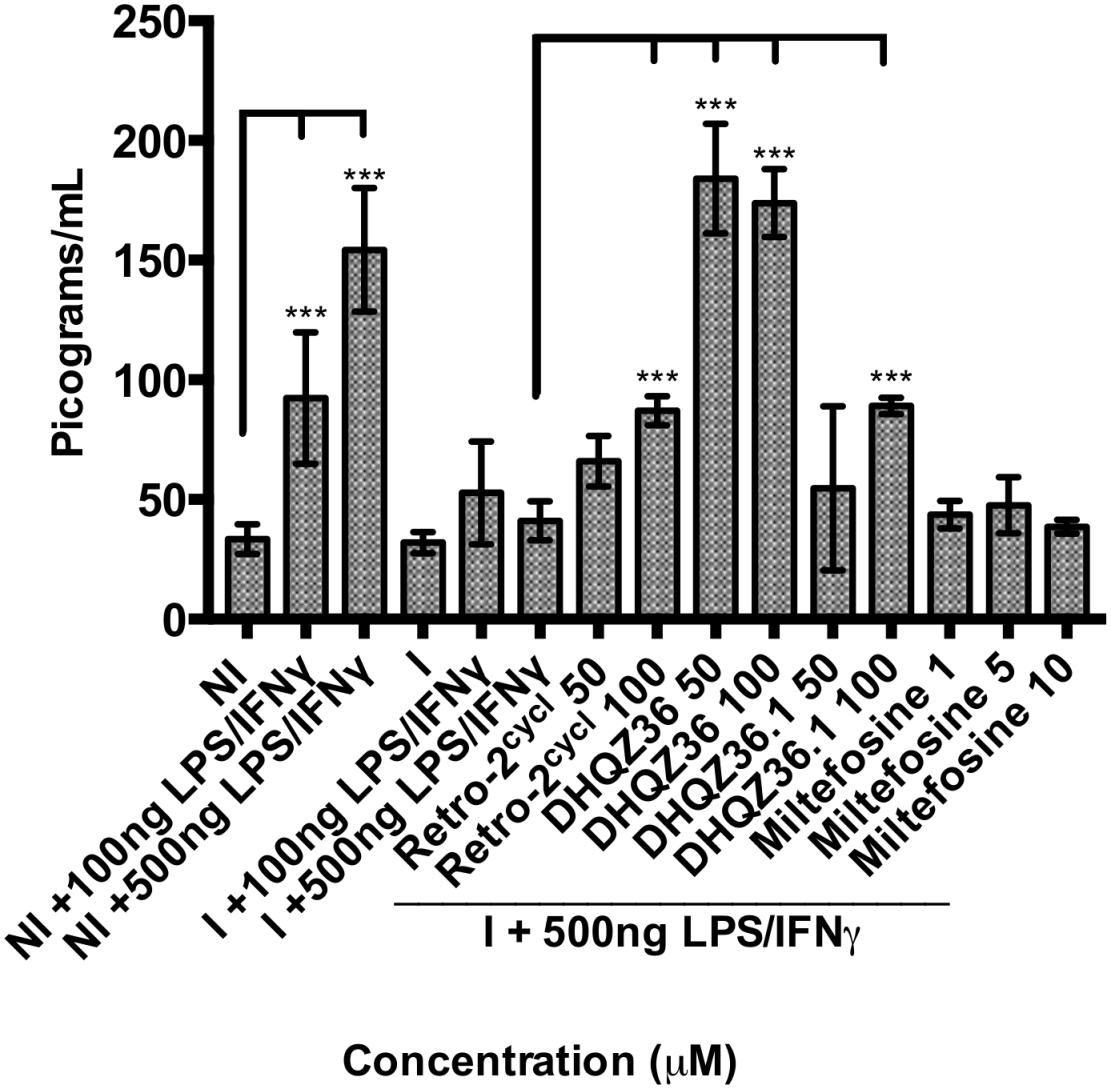
DHQZ analogs relieve IL-6 production by LPS/IFNγ stimulation in *L*. *amazonensis* infected cells. RAW264.7 macrophages were incubated with *L*. *amazonensis* parasites and infections allowed to proceed for 24 hrs. Uninfected or infected cultures were then treated with 100 or 500ng/mL LPS and 100ng/mL IFN-γ. At the same time some cultures where treated with the indicated concentrations of Retro-2^cycl^, DHQZ SAR analogs and miltefosine. After an additional incubation of 24 hrs, the supernatant fluid from each culture was recovered and their IL-6 content determined in an IL-6 specific ELISA. The pg/ml concentration of IL-6 at each drug concentration was compiled from at least three experiments. IL-6 production after drug treatment was compared to comparably activated cultures. Statistical significance between treated cells compared to each control was measured using a two-way ANOVA in GraphPad Prism 7 with the Holm-Sidak posthoc test for multiple comparisons (* = p-value < 0.05, ** = p-value < 0.01, *** p-value < 0.001).

Reduced parasite protein secretion could relieve inhibition of signal transduction schemes that culminate in cytokine release [13][16]. To assess whether Retro-2^cycl^ and the optimized analogs exert any effect on cytokine secretion, macrophage infections were established for 24 hrs, then LPS/IFNγ was added in the presence of increasing concentrations of the DHQZ drugs. Using a cytokine multiplex kit as an initial screen for multiple cytokines, we found that IL-6, especially, was produced by LPS/IFNγ-stimulated infected macrophage cultures after incubation with the DHQZ SAR analogs. To obtain quantification of IL-6 production by infected cultures in response to LPS/IFNγ, IL-6 specific ELISAs were performed. Fig 5 shows data compiled from several ELISA experiments. Incubation of macrophages with LPS/IFNγ results in robust IL-6 production. However, in cultures infected with *L*. *amazonensis* parasites for 24 hrs prior to incubation with LPS/IFNγ, there was significant reduction in the amounts of IL-6 that was produced. This was evidence that parasite infection suppresses IL-6 production in response to LPS/IFNγ. Addition of increasing concentrations of Retro-2^cycl^ resulted in a small but measurable increase in IL-6 production. Similarly, DHQZ 36.1 also resulted in IL-6 production. Most impressively, DHQZ 36 completely reversed the parasite-induced blockade of IL-6 production in response to LPS/IFNγ. Retro-2^cycl^, DHQZ 36 and DHQZ 36.1 by themselves do not induce IL-6 production in uninfected or infected cells without LPS stimulation (S7 Fig; S7 Text). Miltefosine treatment of infected macrophages did not reverse the parasite-induced inhibition of IL-6 production. This is noteworthy in light of the fact that at the higher concentrations of miltefosine (10μM) used in these experiments, approximately 90% of the parasites in macrophages are cleared. Parasite death is evidently not sufficient to lead to the reversal of the inhibitory effects of the parasite on macrophage responses.

## Discussion

Within mammalian cells, *Leishmania* reside in LPVs whose biogenesis and maturation depends in part on the input of molecular components from various host cell compartments. Previous studies had shown that disruption of SNAREs that function in the secretory pathway can limit LPV development [2]. In additional studies, a small compound called Retro-2 that had been shown to block retrograde transport by disrupting Stx5, was shown to limit parasite infections *in vivo* and in macrophages where it reduced the aggrandizement of LPVs that harbored parasites of the *L*. *mexicana* complex [3]. In this study we evaluated two DHQZ SAR analogs that had been found to be more potent than Retro-2 in a DNA virus infection model where virus infection is dependent on retrograde transport [7]. We showed here that in contrast to the parent compound (Retro-2^cycl^), DHQZ 36 and DHQZ 36.1 can kill axenic *Leishmania* promastigotes at low μM concentrations and also promote the clearance of parasites from established macrophage infections. Moreover, these compounds can induce the reversal of the inhibition of IL-6 production by *Leishmania*- infected cells. In light of the fact that the SAR analogs of Retro-2 can kill *Leishmania* parasites directly at low μM concentrations, and that they exhibit a high therapeutic index we propose that they should be considered as suitable candidates for development as drugs to treat *Leishmania* infections.

Retro-2 was identified from a small molecule library screen for compounds that can block ricin toxicity of mammalian cells [5]. Ricin is a prototypic molecule that after engaging lectin receptors on mammalian cells, it is internalized and then traffics through the retrograde transport pathway to the endoplasmic reticulum (ER). From the ER it translocates into the cytosol where it blocks protein synthesis. Like ricin, Shiga toxin produced by *Shigella* is also transported via the retrograde transport route and its toxicity is inhibited by Retro-2 as well. A few viruses including the non-enveloped DNA viruses, Polyomavirus and Papillomavirus are also inhibited by Retro-2 [7]. There is also recent evidence that Retro-2 disrupts vaccinia virus egress from cells [18]. This latter observation suggests that Retro-2 targets a molecular component that is shared by both the retrograde transport machinery and the anterograde transport machinery.

Although the exact mechanism by which Retro-2 disrupts retrograde trafficking is unknown, it clearly induces dispersal of Stx5 from a localized site in the Golgi to more diffuse expression in the cell [3][5]. Recent DHQZ SAR studies have revealed that some SAR analogs can more efficiently block ricin or Shiga toxin transport while others preferentially block transport of papillomavirus and polyomavirus [7][8]. This therefore suggested that there might be subtle but significant differences in the participation of Stx5 in the retrograde transport of various entities.

There are several drugs that are available for the treatment of *Leishmania* infections. Amongst these drugs is miltefosine that was used in comparative experiments in this study. Although the mechanism of miltefosine action in *Leishmania* infections is not known, we are certain that the mechanism of action of the Retro-2^cycl^ and its analogs is different from that of miltefosine. That said, it was valuable to contrast the direct cytolytic effects of miltefosine on *Leishmania* with those of Retro-2^cycl^ and the DHQZ SAR analogs. It is known that *Leishmania* parasites exhibit differential susceptibility to miltefosine [12]. Promastigotes of *L*. *donovani* parasites are more sensitive to miltefosine than promastigotes of *L*. *amazonensis*. Comparable differences in sensitivity to the retrograde trafficking inhibitors were also evident in the studies presented above. Both DHQZ 36 and DHQZ 36.1 were more effective on *L*. *amazonensis* parasites than on *L*. *donovani* parasites. A critical series of experiments revealed that incubation of parasites in either DHQZ 36 or DHQZ 36.1 caused parasite death at low μM concentrations, which in the case of *L*. *amazonensis* was at concentrations lower than miltefosine. Parasites that were treated with the retrograde trafficking inhibitors and transferred to fresh media did not recover from the physiological perturbations caused by the compounds. This was in contrast to incubation in Retro-2^cycl^ from which parasites recovered robustly after removal from the drug, including at concentrations that were much higher than the drug EC_50_. *L*. *amazonensis* parasites recovered from incubation with miltefosine at concentrations that were close to the drug EC_50_ on these parasites. Although the EC_50_ for axenic parasite clearance is much higher than the drug concentrations that are required to kill intracellular parasites, it is quite likely that drugs that cannot kill parasites directly might be less effective in clearing parasites in an infected host.

The effect of Retro-2 and the SAR analogs on IL-6 production by infected cells activated with LPS and IFN-γ was intriguing. Several reports have shown that macrophages infected by several species of *Leishmania* are refractory to LPS induced cytokine production [13][14][15]. The underlying mechanism(s) by which parasites within cells suppress cytokine production is not known. Some of the mechanisms that have been proposed have included the likelihood that parasite proteins are exported from the LPV into the cell cytosol (possible traffic to the nucleus as well) where they encounter and disrupt signal transduction that would otherwise lead to the production of cytokines. We had shown previously that Retro-2 does not alter secretion of macrophage molecules in non-infected cells [3]. In light of the fact that *Leishmania* parasites have only 27 SNARE genes as compared to mice and man that have over 38 SNARE genes it has been proposed that parasites would be more sensitive to the disruption of the function of any individual SNAREs [19][20]. We considered the likelihood that Retro-2’s actions in the parasite could include disruption of secretion. Some evidence that protein secretion by parasites is affected by the Retro-2 SAR analogs is shown in supplement 1. A Click-iT protocol was employed to evaluate general secretion; there is reduced secretion in the presence of DHQZ 36 and DHQZ 36.1 than with Retro-2^cycl^ or miltefosine. The alternative explanation for the DHQZs induction of the reversal of IL-6 production after LPS activation is that *Leishmania* infection might lead to disruption of IL-6 trafficking through secretory compartments. Taken together, in addition to being useful therapeutic agents to control leishmaniasis, the structurally optimized retrograde trafficking inhibitors could also prove to be valuable reagents for the dissection of cytokine trafficking in *Leishmania*-infected cells.

## Supporting information

S1 Fig Synthesis scheme of DHQZ 36 and DHQZ 36.1 Scheme. Reagents and conditions: (a) EDC, DMAP, dichloromethane were combined at room temperature, 16 h; (b) thereafter 10% Pd/C, ammonium formate, methanol, were added at room temperature, 2 h, 50% over two steps; (c) 5-ethylthiophene-2-carboxaldehyde or 5-(2-methylthiazole)-thiophene-2-carboxaldehyde Sc(OTf)3, methanol, MW irradiation, 100°C, 1 h, 75–89%.
(TIFF)

S2 Fig Spectra of DHQZ36. DHQZ 36 was isolated as an off white/yellow solid (259 mg, 0.75 mmol, 75% yield). NMR ^1^H (400 MHz, CDCl_3_) δ 7.72 (dd, *J* = 8.8, 2.9 Hz, 1 H), 7.32–7.22 (m, 3 H), 7.10–6.99 (m, 3 H), 6.74 (d, *J* = 3.4 Hz, 1 H), 6.63–6.55 (m, 2 H), 5.74 (s, 1 H), 5.51 (d, *J* = 15.2 Hz, 1 H), 3.87 (d, *J* = 15.2 Hz, 1 H), 3.87 (d, *J* = 15.2 Hz, 1 H), 2.75 (q, *J* = 7.5 Hz, 2 H), 1.25 (t, *J* = 7.6 Hz, 3H) (A). ^13^C (100 MHz, CDCl_3_) δ163.5, 161.8, 155.7, 148.9, 141.0, 139.3, 132.4, 129.7, 126.3, 122.5, 121.1, 117.3, 116.4, 115.6, 114.6, 67.3, 46.4, 23.5, 15.7. HRMS (ESI): m/z calcd for C_21_H_18_F_2_N_2_OS [M+H]^+^: 385.1181, found: 385.1185 (B).
(TIFF)

S3 Fig Spectra of DHQZ36.1. DHQZ36.1 was isolated as a yellow solid (203 mg, 0.447 mmol, 89% yield). NMR ^1^H (400 MHz, CDCl_3_) δ: 7.71 (dd, *J* = 8.8, 2.9 Hz, 1 H), 7.33–7.24 (m, 3 H), 7.21 (d, *J* = 3.7 Hz, 1 H), 7.15 (s, 1 H), 7.08–6.98 (m, 3 H), 6.85 (d, *J* = 3.8 Hz, 1 H), 6.60 (dd, *J* = 8.7, 4.2 Hz, 1 H), 5.78 (s, 1 H), 5.57 (d, *J* = 15.3 Hz, 1 H), 3.88 (d, *J* = 15.3 Hz, 1 H), 2.73 (s, 3 H) (A). ^13^C (100 MHz, CDCl_3_) δ 166.7, 163.6, 161.7, 161.1, 158.2, 155.8, 148.5, 141.9, 140.8, 138.8, 132.2, 129.8, 127.0, 123.2, 121.2, 117.3, 116.7, 115.7, 114.6, 112.0, 66.9, 46.5, 19.1. HRMS (ESI): mz/ calcd for C_23_H_17_F_2_N_3_OS_2_ [M+H]^+^: 454.0854, found: 454.0858 (B).
(TIFF)

S4 Fig Evaluation of toxicity of Retro-2 and analogs. RAW264.7 macrophages were plated for 24 hours and treated with Retro-2^cycl^ or DHQZ compounds for 24 hours. Supernatants were taken and tested in triplicate for LDH release and measured as % cell viability as compared to the maximum LDH released after subtraction of the media control. Error bars are shown as standard deviation.
(TIFF)

S5 Fig Nitric oxide production by *L*. *amazonensis*-infected RAW 264.7 cells. A) RAW264.7 macrophages infected for 24 hrs or uninfected were treated with Retro-2^cycl^, or DHQZ 36 or DHQZ 36.1 or miltefosine at the indicated concentrations. The cell supernatant fluid from each culture was recovered after 24 hrs. The production of nitric oxide was quantified using the Greiss reagent. [Note. The plot includes nitric oxide levels from the LPS/IFNγ treated uninfected and infected samples to provide a reference of the nitrite levels in unstimulated cultures]. B) Uninfected and infected cultures were activated with 500 ng/mL LPS and 100 ng/mL IFNγ to these cultures the indicated concentrations of Retro-2^cycl^ or DHQZ 36 or DHQZ 36.1 or miltefosine were added. Nitric oxide in the supernatant was measured after 24 hrs culture. Supernatants were tested in triplicate and significance was measured using the GraphPad Prism 7 Student’s t-test with and p-values are noted as * < 0.05, ** < 0.01 and *** < 0.001. This is representative of two experiments.
(TIFF)

S6 Fig Retro-2 analogs suppress protein secretion by promastigotes cultures. Parasite cultures metabolically labeled with L-azidohomoalaine (AHA) were incubated with the indicated μM amounts of Retro-2 or its analogs or with miltefosine. After biotinylation of culture supernatants with Click Chemistry, the samples were analyzed by Western blotting and probed with avidin-HRP (A). Control cells were run for comparison. A densitometric scan of the prominent bands was obtained (B). This figure is representative of two experiments.
(TIFF)

S7 Fig IL-6 production by infected RAW264.7 macrophages without LPS activation. Macrophages were incubated with *L*. *amazonensis* parasites and infections allowed to proceed for 24hrs. Infected cultures were then treated with the indicated concentrations of Retro-2^cycl^, DHQZ SAR analogs and Miltefosine without LPS activation. After an additional incubation of 24hrs, the supernatant fluid from each culture was recovered and their IL-6 content determined in an IL-6 specific ELISA. The pg/ml concentration of IL-6 at each drug concentration was compiled from at least three experiments. Experiments were run in duplicate. IL-6 production after drug treatment was compared to comparably activated cultures. (NI denotes non-infected macrophages) Statistical significance between treated cells compared to each control was measured using a two-way ANOVA in GraphPad Prism 7 with the Tukey posthoc test for multiple comparisons (* = p-value < 0.05, ** = p-value < 0.01, *** p-value < 0.001).
(TIFF)

S1 Text Synthesis of DHQZ 36 and DHQZ 36.1. Preparation of 5-fluoro-N-(4-fluorobenzyl)-2-nitrobenzamide (**S1**): In a clean, dry round bottom flask 5-fluoro-2-nitro benzoic acid (407 mg, 2.2 mmol, 1.1 equiv.), 1-ethyl-3-(3-dimethylaminopropyl)carbodiimide HCl (422 mg, 2.2 mmol, 1.1 equiv), hydroxybenzotriazole hydrate (337 mg, 2.2 mmol, 1.1 equiv), and dimethylaminopyridine (24 mg, 0.2 mmol, 0.1 equiv) was dissolved in 10 mL dichloromethane (DCM). To this was added 4-fluorobenzylamine (229 μL, 2.0 mmol, 1 equiv) and reaction was allowed to stir at room temperature for 16 hours. Reaction was diluted with DCM and extracted successively with aqueous solutions of 1M HCl, saturated sodium bicarbonate, and brine. DCM was dried over sodium sulfate and removed under reduced pressure. Residue was purified on a silica column using a 2:1 Hexanes: Ethyl Acetate solvent system to yield **S1** as a white solid in 96% yield (560 mg, 1.92 mmol). Spectral data was consistent with published structures.
Preparation of **DHQZ 36** and **DHQZ 36.1**
**Step 1:** To a solution of **S1** (560 mg, 1.92 mmol, 1 equiv) in 10 mL methanol was added ammonium formate (133 mg, 2.1 mmol, 1.1 equiv) and 300 mg 10% Pd-C. Reaction was stirred at room temperature for about 2 hours. Once reaction was complete as assessed by TLC, Pd-C was filtered through celite and methanol was removed under reduced pressure. The residue was purified via column chromatography using a gradient of 20% ethyl acetate in hexanes to yield the pure amine (261 mg, 1 mmol, 52% yield). **Step 2:** The amine was dissolved in 3 mL methanol in a microwave vial. To this, 0.1 equivalent scandium triflate and 1.1 equivalent of the corresponding aldehyde was added. The vial was sealed and subjected to microwave irradiation at 100°C for 1 hour. Methanol was removed under reduced pressure and the residue was purified on a silica column using a gradient of ethyl acetate in hexanes.
(PDF)

S2 Text DHQZ 36 was isolated as an off white/yellow solid (259 mg, 0.75 mmol, 75% yield). NMR ^1^H (400 MHz, CDCl_3_) δ 7.72 (dd, *J* = 8.8, 2.9 Hz, 1 H), 7.32–7.22 (m, 3 H), 7.10–6.99 (m, 3 H), 6.74 (d, *J* = 3.4 Hz, 1 H), 6.63–6.55 (m, 2 H), 5.74 (s, 1 H), 5.51 (d, *J* = 15.2 Hz, 1 H), 3.87 (d, *J* = 15.2 Hz, 1 H), 3.87 (d, *J* = 15.2 Hz, 1 H), 2.75 (q, *J* = 7.5 Hz, 2 H), 1.25 (t, *J* = 7.6 Hz, 3H) (A). ^13^C (100 MHz, CDCl_3_) δ163.5, 161.8, 155.7, 148.9, 141.0, 139.3, 132.4, 129.7, 126.3, 122.5, 121.1, 117.3, 116.4, 115.6, 114.6, 67.3, 46.4, 23.5, 15.7. HRMS (ESI): m/z calcd for C_21_H_18_F_2_N_2_OS [M+H]^+^: 385.1181, found: 385.1185 (B).
(PDF)

S3 Text DHQZ36.1 was isolated as a yellow solid (203 mg, 0.447 mmol, 89% yield). NMR ^1^H (400 MHz, CDCl_3_) δ: 7.71 (dd, *J* = 8.8, 2.9 Hz, 1 H), 7.33–7.24 (m, 3 H), 7.21 (d, *J* = 3.7 Hz, 1 H), 7.15 (s, 1 H), 7.08–6.98 (m, 3 H), 6.85 (d, *J* = 3.8 Hz, 1 H), 6.60 (dd, *J* = 8.7, 4.2 Hz, 1 H), 5.78 (s, 1 H), 5.57 (d, *J* = 15.3 Hz, 1 H), 3.88 (d, *J* = 15.3 Hz, 1 H), 2.73 (s, 3 H) (A). ^13^C (100 MHz, CDCl_3_) δ 166.7, 163.6, 161.7, 161.1, 158.2, 155.8, 148.5, 141.9, 140.8, 138.8, 132.2, 129.8, 127.0, 123.2, 121.2, 117.3, 116.7, 115.7, 114.6, 112.0, 66.9, 46.5, 19.1. HRMS (ESI): mz/ calcd for C_23_H_17_F_2_N_3_OS_2_ [M+H]^+^: 454.0854, found: 454.0858 (B).
(PDF)

S4 Text LDH Assay RAW264.7 macrophages were seeded at 2000 cells/well in 96-well plates at 37°C in 5% CO_2_ overnight for adherence. Macrophages were then treated with DMSO (vehicle) or DHQZ compounds at concentration ranges of 5–300 μM. For the Max LDH estimation and Spontaneous LDH control, wells were treated with Lysis Buffer or sterile diH_2_O and incubated for 45 minutes at 37°C for lysis to occur. Plates were spun down and supernatant was collected and measured for LDH at 490 nm and 580 nm background as described in the Pierce LDH Cytotoxicity Assay Kit protocol (ThermoFisher Scientific). DHQZ compound and miltefosine treated cells were compared to the lysis buffer Max LDH control to determine % cytotoxicity. Background was measured and subtracted from wells by subtracting a media control with or without lysis buffer as well as the Spontaneous LDH control.
(PDF)

S5 Text Griess reaction. RAW264.7 macrophages were seeded at 3x10^5^ cells/well over glass coverslips in 6-well plates overnight for adherence. Macrophages were then infected with stationary *L*. *amazonensis* promastigotes for 24 hours before treatment with Retro-2cycl, DHQZ compounds or miltefosine. For assessment of stimulated macrophages 500 ng/mL LPS and 100 ng/mL IFNγ were added in addition to the drugs and incubated for an additional 24 hours. A replicate set of wells were treated with 10 μM N-acetyl-L-cysteine (NALC) as a superoxide scavenger. Non-infected, infected and infected cells treated with NALC were treated for 24 hours before supernatants were collected and spun down to remove any cellular particles. Supernatants were tested in triplicate for nitrite concentration using the Invitrogen Griess Reagent Kit protocol (ThermoFisher Scientific).
(PDF)

S6 Text Protocol. Promastigotes in early stationary phase were seeded at 5x10^8^ parasites/mL in methionine-free DMEM medium purchased from ThermoFisher and supplemented with 2% dialyzed FBS and 1% penicillin-streptomycin. After 1 hr of methionine starvation, cells were spun down and resuspended in fresh met-free DMEM supplemented with 100μM L-Azidohomoalanine (AHA). Parasites were incubated with AHA supplemented medium for 5 hrs before resuspension in 1% FBS, 1% penicillin-streptomycin Schneider’s medium containing DHQZ analogs or miltefosine at low or high concentrations for 6 hrs. After 6 hrs, cells were spun down and supernatants were collected and treated with 0.1% Triton X-100 for 30 minutes. Aliquots of the supernatant fluid were then frozen at -20°C overnight. They were concentrated by methanol-chloroform precipitation and resuspended in 50mM Tris-HCl, pH 8.0, with 1% SDS as described in the Click-iT Metabolic Labeling Reagents for Proteins protocol for biotinylation (ThermoFisher). Proteins in the suspension were Click-labeled using alkyne biotin purchased from ThermoFisher (Molecular Probes; B10185) and the Click-iT Protein Reaction Buffer Kit (Molecular Probes; C10276). Proteins were then run on Mini-PROTEAN TGX Stain-Free gels purchased from Bio-Rad (Cat: 456–8104) and transferred to nitrocellulose membranes following normal Western blotting procedures. Membranes were blocked in 5% BSA overnight and Click-labeled proteins were detected using a 30 minutes incubation with streptavidin-HRP at a 1:2500 dilution in 2% BSA followed by ECL Western substrate.
(PDF)
