# Supplementary figures and images for: Structurally optimized analogs of the retrograde trafficking inhibitor Retro-2cycl limit Leishmania infections
Source: PLoS Negl Trop Dis. 2017 May 15;11(5):e0005556. doi: 10.1371/journal.pntd.0005556 (PMC5444862; doi:10.1371/journal.pntd.0005556)

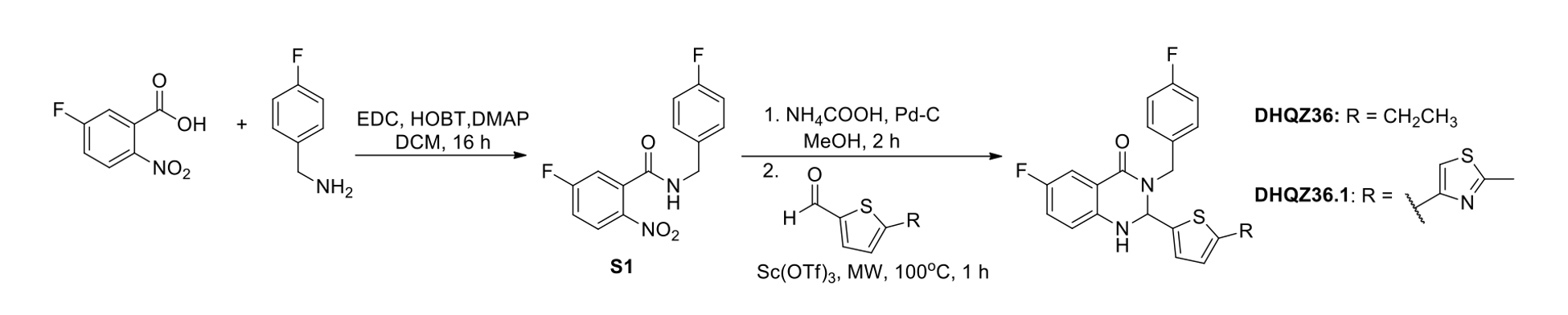

Supplement: S1 Fig — Reagents and conditions: (a) EDC, DMAP, dichloromethane were combined at room temperature, 16 h; (b) thereafter 10% Pd/C, ammonium formate, methanol, were added at room temperature, 2 h, 50% over two steps; (c) 5-ethylthiophene-2-carboxaldehyde or 5-(2-methylthiazole)-thiophene-2-carboxaldehyde Sc(OTf)3, methanol, MW irradiation, 100°C, 1 h, 75–89%. (TIFF) [file pntd.0005556.s001.tiff]

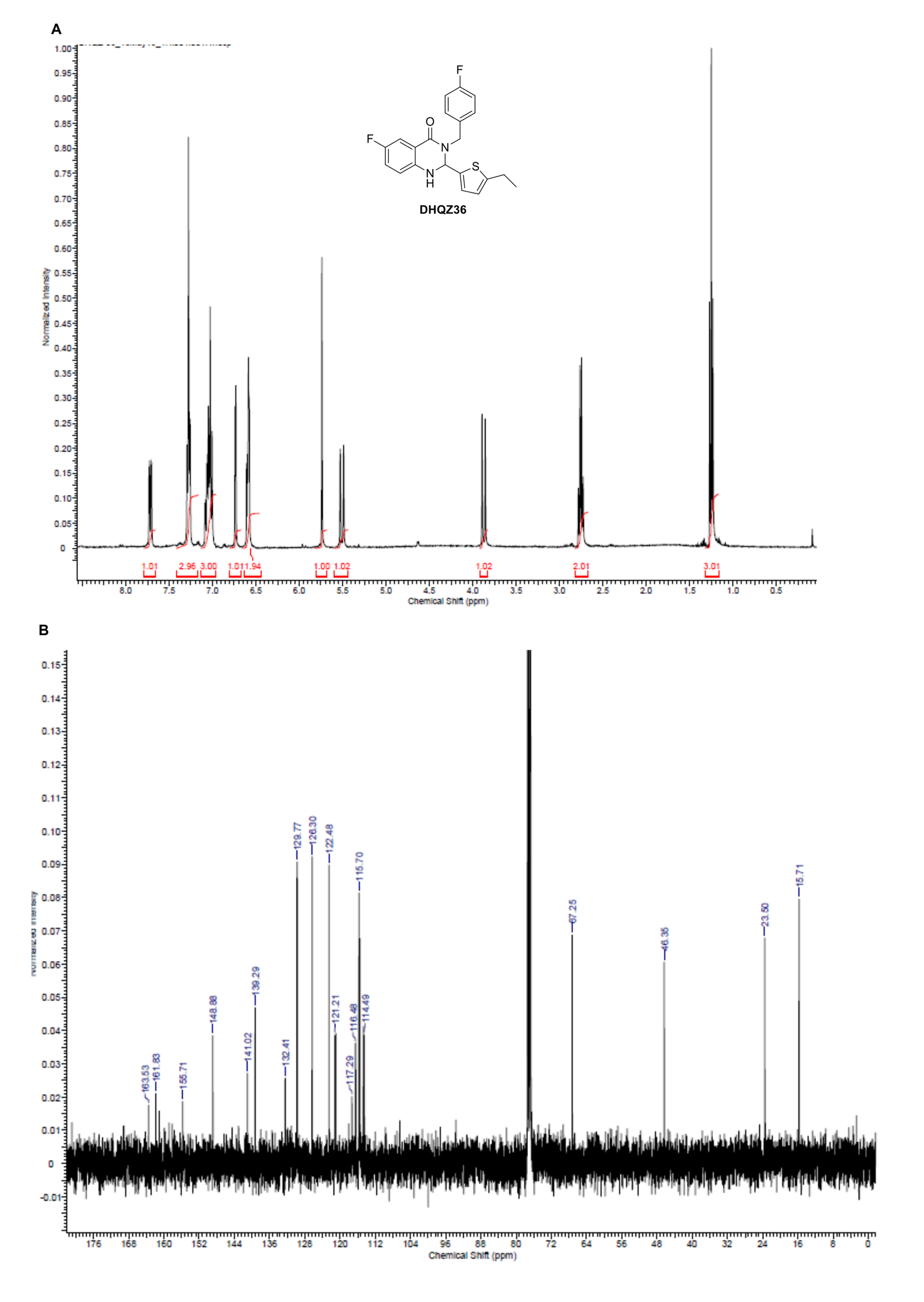

Supplement: S2 Fig — NMR 1H (400 MHz, CDCl3) δ 7.72 (dd, J = 8.8, 2.9 Hz, 1 H), 7.32–7.22 (m, 3 H), 7.10–6.99 (m, 3 H), 6.74 (d, J = 3.4 Hz, 1 H), 6.63–6.55 (m, 2 H), 5.74 (s, 1 H), 5.51 (d, J = 15.2 Hz, 1 H), 3.87 (d, J = 15.2 Hz, 1 H), 3.87 (d, J = 15.2 Hz, 1 H), 2.75 (q, J = 7.5 Hz, 2 H), 1.25 (t, J = 7.6 Hz, 3H) (A). 13C (100 MHz, CDCl3) δ163.5, 161.8, 155.7, 148.9, 141.0, 139.3, 132.4, 129.7, 126.3, 122.5, 121.1, 117.3, 116.4, 115.6, 114.6, 67.3, 46.4, 23.5, 15.7. HRMS (ESI): m/z calcd for C21H18F2N2OS [M+H]+: 385.1181, found: 385.1185 (B). (TIFF) [file pntd.0005556.s002.tiff]

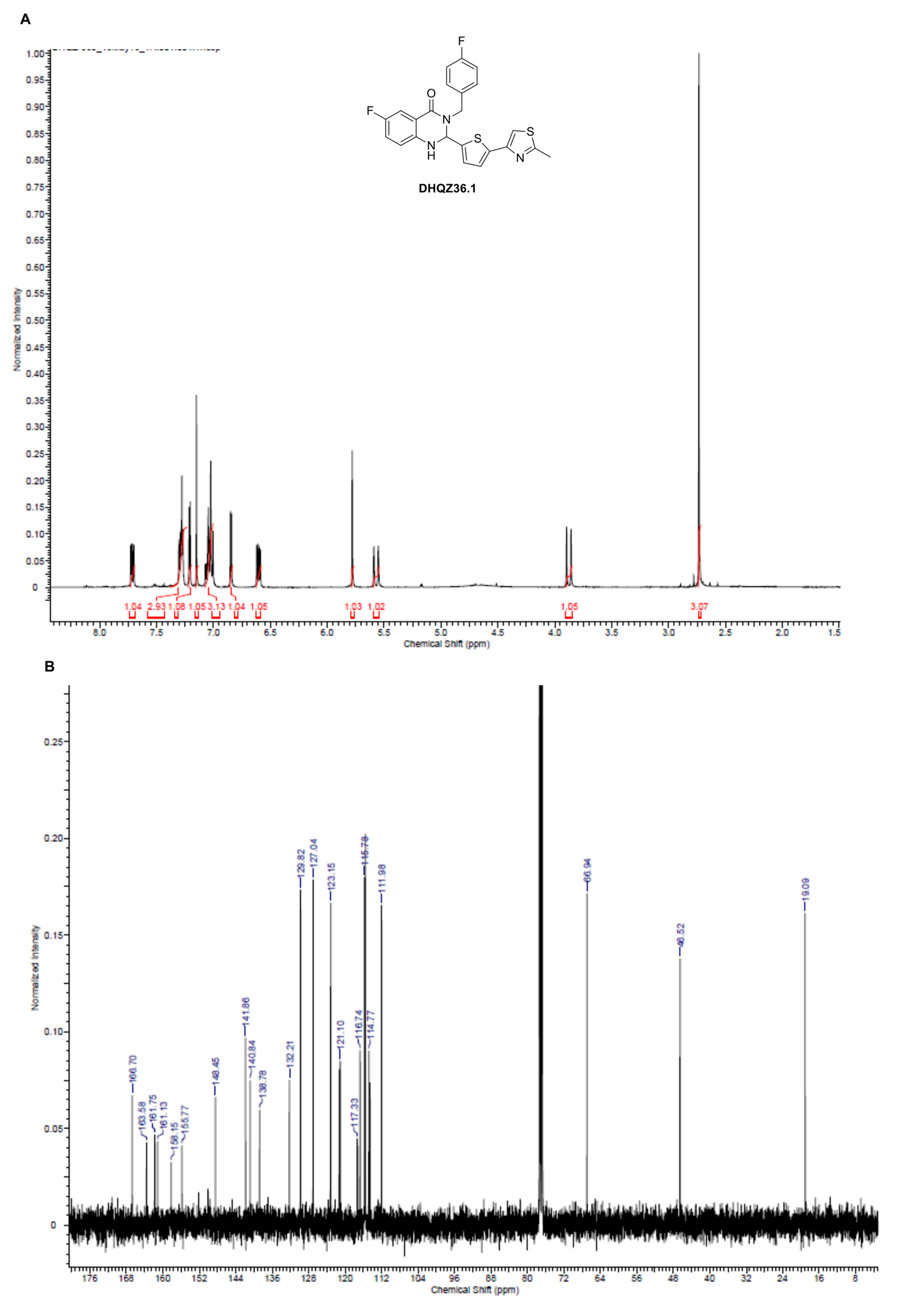

Supplement: S3 Fig — NMR 1H (400 MHz, CDCl3) δ: 7.71 (dd, J = 8.8, 2.9 Hz, 1 H), 7.33–7.24 (m, 3 H), 7.21 (d, J = 3.7 Hz, 1 H), 7.15 (s, 1 H), 7.08–6.98 (m, 3 H), 6.85 (d, J = 3.8 Hz, 1 H), 6.60 (dd, J = 8.7, 4.2 Hz, 1 H), 5.78 (s, 1 H), 5.57 (d, J = 15.3 Hz, 1 H), 3.88 (d, J = 15.3 Hz, 1 H), 2.73 (s, 3 H) (A). 13C (100 MHz, CDCl3) δ 166.7, 163.6, 161.7, 161.1, 158.2, 155.8, 148.5, 141.9, 140.8, 138.8, 132.2, 129.8, 127.0, 123.2, 121.2, 117.3, 116.7, 115.7, 114.6, 112.0, 66.9, 46.5, 19.1. HRMS (ESI): mz/ calcd for C23H17F2N3OS2 [M+H]+: 454.0854, found: 454.0858 (B). (TIFF) [file pntd.0005556.s003.tiff]

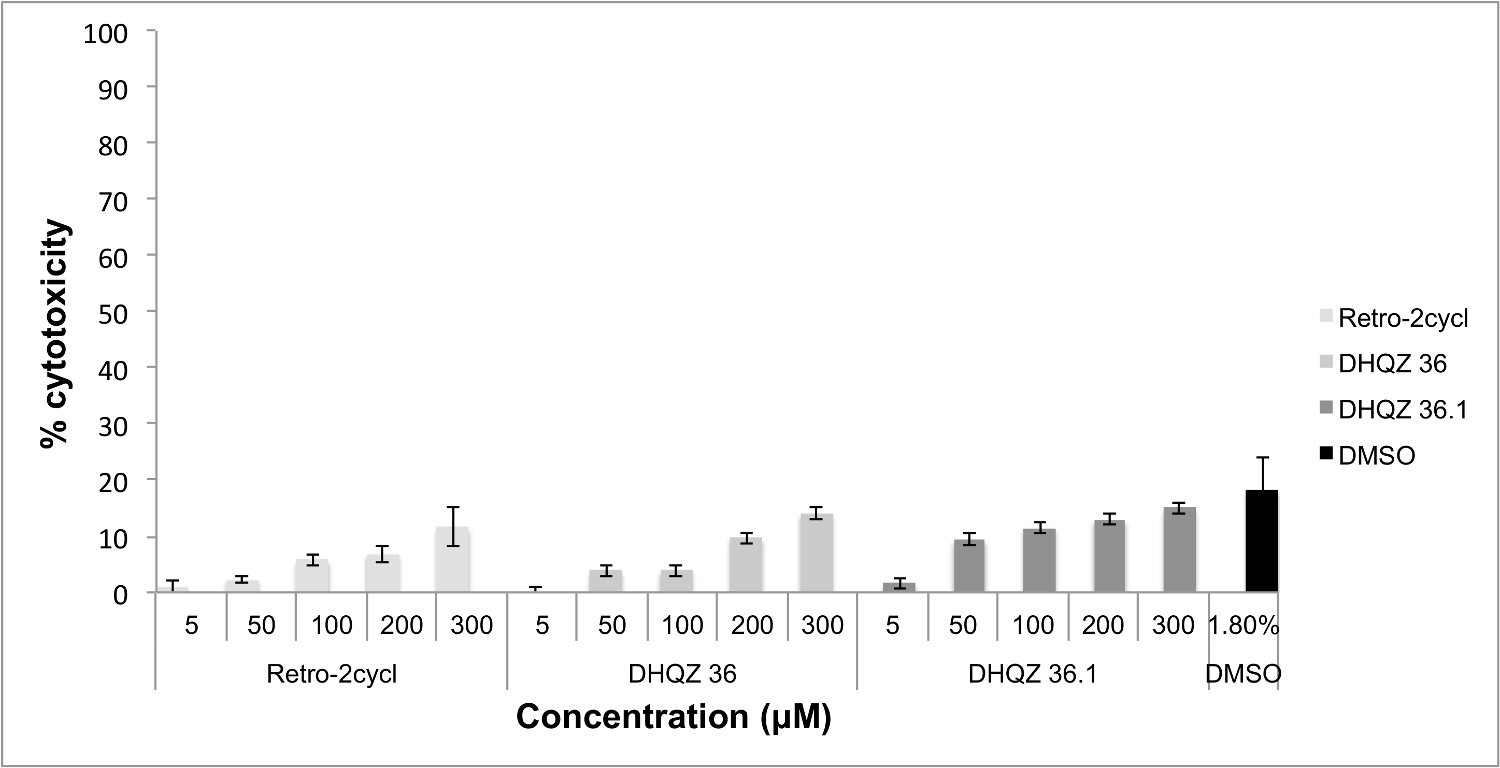

Supplement: S4 Fig — RAW264.7 macrophages were plated for 24 hours and treated with Retro-2cycl or DHQZ compounds for 24 hours. Supernatants were taken and tested in triplicate for LDH release and measured as % cell viability as compared to the maximum LDH released after subtraction of the media control. Error bars are shown as standard deviation. (TIFF) [file pntd.0005556.s004.tiff]

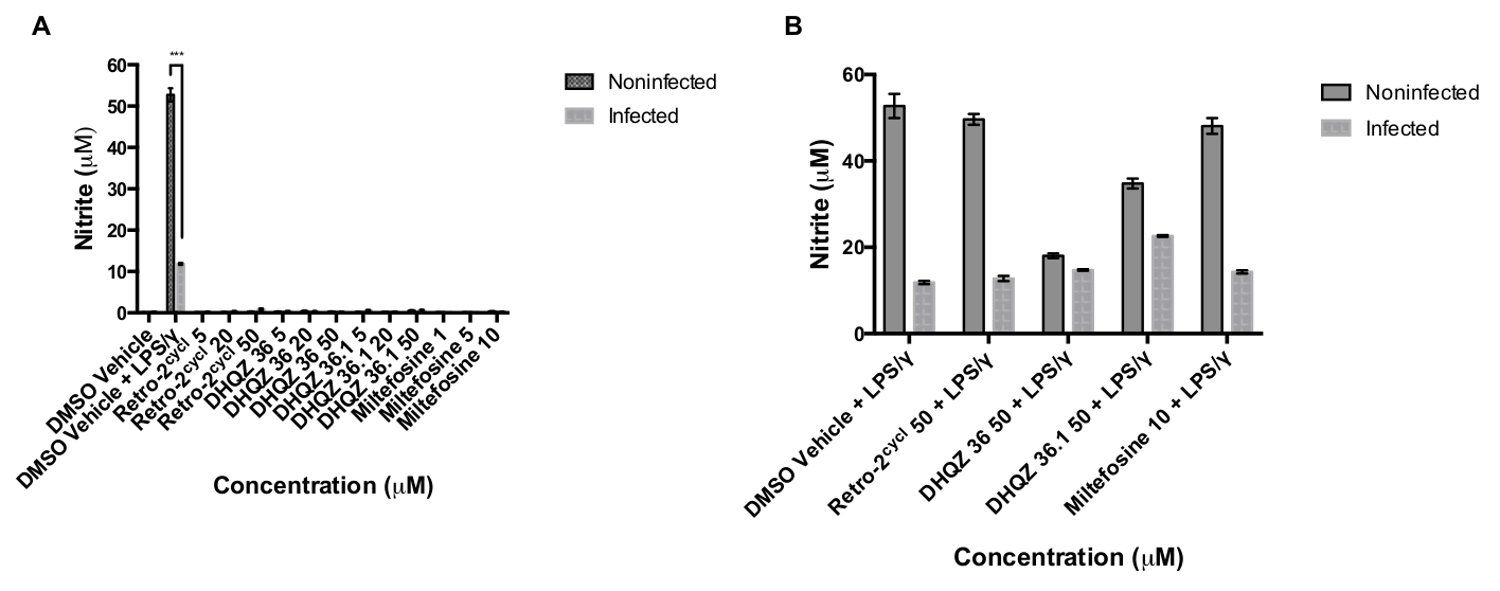

Supplement: S5 Fig — A) RAW264.7 macrophages infected for 24 hrs or uninfected were treated with Retro-2cycl, or DHQZ 36 or DHQZ 36.1 or miltefosine at the indicated concentrations. The cell supernatant fluid from each culture was recovered after 24 hrs. The production of nitric oxide was quantified using the Greiss reagent. [Note. The plot includes nitric oxide levels from the LPS/IFNγ treated uninfected and infected samples to provide a reference of the nitrite levels in unstimulated cultures]. B) Uninfected and infected cultures were activated with 500 ng/mL LPS and 100 ng/mL IFNγ to these cultures the indicated concentrations of Retro-2cycl or DHQZ 36 or DHQZ 36.1 or miltefosine were added. Nitric oxide in the supernatant was measured after 24 hrs culture. Supernatants were tested in triplicate and significance was measured using the GraphPad Prism 7 Student’s t-test with and p-values are noted as * < 0.05, ** < 0.01 and *** < 0.001. This is representative of two experiments. (TIFF) [file pntd.0005556.s005.tiff]

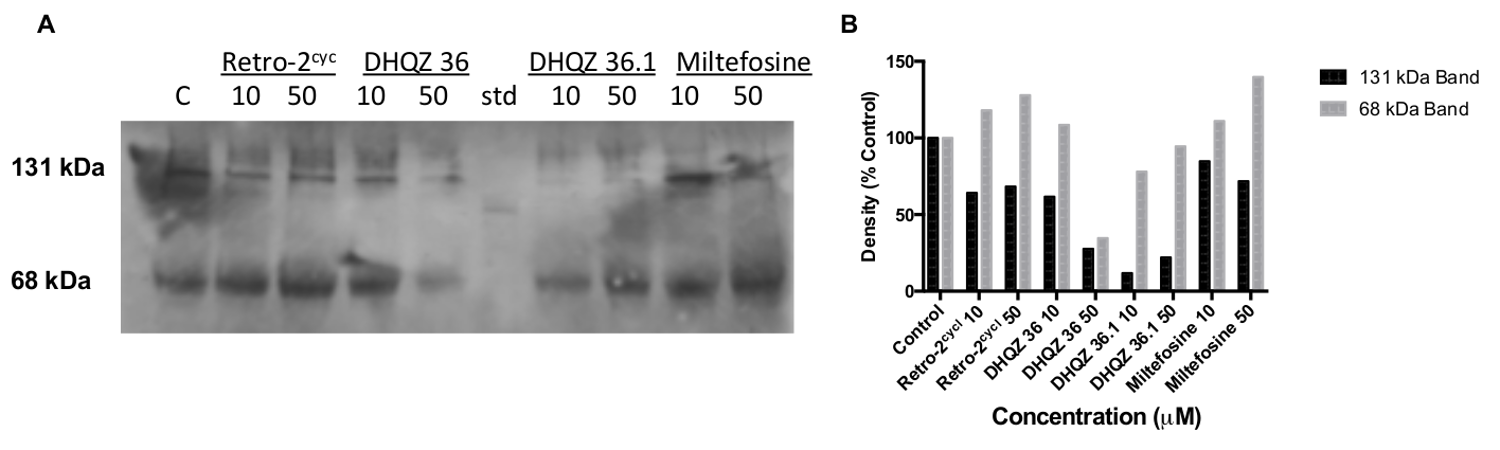

Supplement: S6 Fig — Parasite cultures metabolically labeled with L-azidohomoalaine (AHA) were incubated with the indicated μM amounts of Retro-2 or its analogs or with miltefosine. After biotinylation of culture supernatants with Click Chemistry, the samples were analyzed by Western blotting and probed with avidin-HRP (A). Control cells were run for comparison. A densitometric scan of the prominent bands was obtained (B). This figure is representative of two experiments. (TIFF) [file pntd.0005556.s006.tiff]

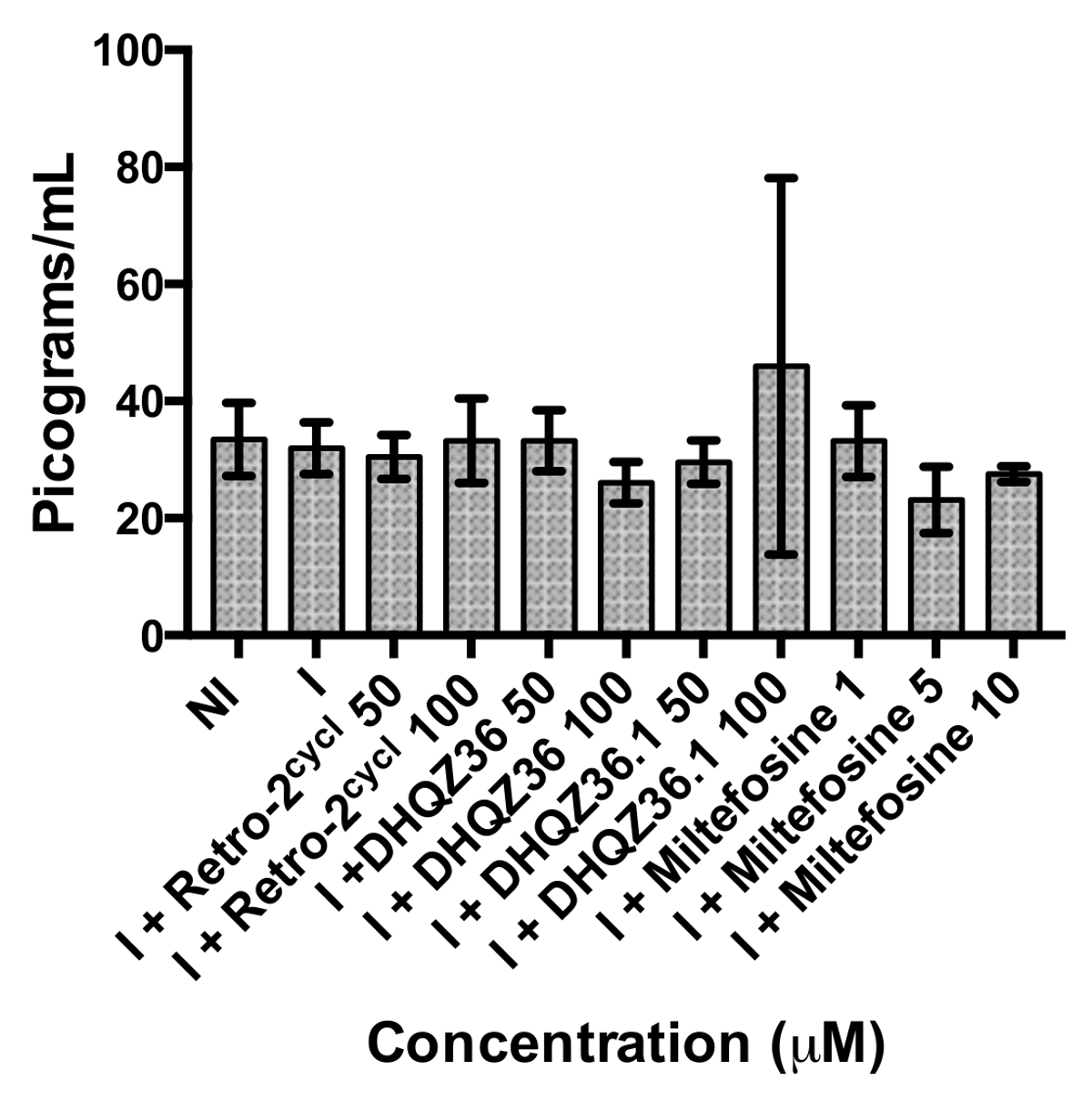

Supplement: S7 Fig — Macrophages were incubated with L. amazonensis parasites and infections allowed to proceed for 24hrs. Infected cultures were then treated with the indicated concentrations of Retro-2cycl, DHQZ SAR analogs and Miltefosine without LPS activation. After an additional incubation of 24hrs, the supernatant fluid from each culture was recovered and their IL-6 content determined in an IL-6 specific ELISA. The pg/ml concentration of IL-6 at each drug concentration was compiled from at least three experiments. Experiments were run in duplicate. IL-6 production after drug treatment was compared to comparably activated cultures. (NI denotes non-infected macrophages) Statistical significance between treated cells compared to each control was measured using a two-way ANOVA in GraphPad Prism 7 with the Tukey posthoc test for multiple comparisons (* = p-value < 0.05, ** = p-value < 0.01, *** p-value < 0.001). (TIFF) [file pntd.0005556.s007.tiff]
