# Supplementary material for: Structurally optimized analogs of the retrograde trafficking inhibitor Retro-2cycl limit Leishmania infections
Source: PLoS Negl Trop Dis. 2017 May 15;11(5):e0005556. doi: 10.1371/journal.pntd.0005556 (PMC5444862; doi:10.1371/journal.pntd.0005556)
Supplement: S1 Text — Preparation of 5-fluoro-N-(4-fluorobenzyl)-2-nitrobenzamide (S1): In a clean, dry round bottom flask 5-fluoro-2-nitro benzoic acid (407 mg, 2.2 mmol, 1.1 equiv.), 1-ethyl-3-(3-dimethylaminopropyl)carbodiimide HCl (422 mg, 2.2 mmol, 1.1 equiv), hydroxybenzotriazole hydrate (337 mg, 2.2 mmol, 1.1 equiv), and dimethylaminopyridine (24 mg, 0.2 mmol, 0.1 equiv) was dissolved in 10 mL dichloromethane (DCM). To this was added 4-fluorobenzylamine (229 μL, 2.0 mmol, 1 equiv) and reaction was allowed to stir at room temperature for 16 hours. Reaction was diluted with DCM and extracted successively with aqueous solutions of 1M HCl, saturated sodium bicarbonate, and brine. DCM was dried over sodium sulfate and removed under reduced pressure. Residue was purified on a silica column using a 2:1 Hexanes: Ethyl Acetate solvent system to yield S1 as a white solid in 96% yield (560 mg, 1.92 mmol). Spectral data was consistent with published structures. Preparation of DHQZ 36 and DHQZ 36.1 Step 1: To a solution of S1 (560 mg, 1.92 mmol, 1 equiv) in 10 mL methanol was added ammonium formate (133 mg, 2.1 mmol, 1.1 equiv) and 300 mg 10% Pd-C. Reaction was stirred at room temperature for about 2 hours. Once reaction was complete as assessed by TLC, Pd-C was filtered through celite and methanol was removed under reduced pressure. The residue was purified via column chromatography using a gradient of 20% ethyl acetate in hexanes to yield the pure amine (261 mg, 1 mmol, 52% yield). Step 2: The amine was dissolved in 3 mL methanol in a microwave vial. To this, 0.1 equivalent scandium triflate and 1.1 equivalent of the corresponding aldehyde was added. The vial was sealed and subjected to microwave irradiation at 100°C for 1 hour. Methanol was removed under reduced pressure and the residue was purified on a silica column using a gradient of ethyl acetate in hexanes. (PDF) [file pntd.0005556.s008.pdf]

SI Text. Synthesis of DHQZ 36 and DHQZ 36.1. Preparation of 5-fluoro-N-(4-fluorobenzyl)-2-nitrobenzamide (**S1**): In a clean, dry round bottom flask 5-fluoro-2-nitro benzoic acid (407 mg, 2.2 mmol, 1.1 equiv.), 1-ethyl-3-(3-dimethylaminopropyl)carbodiimide HCl (422 mg, 2.2 mmol, 1.1 equiv), hydroxybenzotriazole hydrate (337 mg, 2.2 mmol, 1.1 equiv), and dimethylaminopyridine (24 mg, 0.2 mmol, 0.1 equiv) was dissolved in 10 mL dichloromethane (DCM). To this was added 4-fluorobenzylamine (229 μL, 2.0 mmol, 1 equiv) and reaction was allowed to stir at room temperature for 16 hours. Reaction was diluted with DCM and extracted successively with aqueous solutions of 1M HCl, saturated sodium bicarbonate, and brine. DCM was dried over sodium sulfate and removed under reduced pressure. Residue was purified on a silica column using a 2:1 Hexanes: Ethyl Acetate solvent system to yield **S1** as a white solid in 96% yield (560 mg, 1.92 mmol). Spectral data was consistent with published structures.[1]

Preparation of **DHQZ 36** and **DHQZ 36.1**

Step 1: To a solution of **S1** (560 mg, 1.92 mmol, 1 equiv) in 10 mL methanol was added ammonium formate (133 mg, 2.1 mmol, 1.1 equiv) and 300 mg 10% Pd-C. Reaction was stirred at room temperature for about 2 hours. Once reaction was complete as assessed by TLC, Pd-C was filtered through celite and methanol was removed under reduced pressure. The residue was purified via column chromatography using a gradient of 20% ethyl acetate in hexanes to yield the pure amine (261 mg, 1 mmol, 52% yield). **Step 2:** The amine was dissolved in 3 mL methanol in a microwave vial. To this, 0.1 equivalent scandium triflate and 1.1 equivalent of the corresponding aldehyde was added. The vial was sealed and subjected to microwave irradiation at 100°C for 1

hour. Methanol was removed under reduced pressure and the residue was purified on a silica column using a gradient of ethyl acetate in hexanes.
