# Supplementary material for: Structurally optimized analogs of the retrograde trafficking inhibitor Retro-2cycl limit Leishmania infections
Source: PLoS Negl Trop Dis. 2017 May 15;11(5):e0005556. doi: 10.1371/journal.pntd.0005556 (PMC5444862; doi:10.1371/journal.pntd.0005556)
Supplement: S2 Text — NMR 1H (400 MHz, CDCl3) δ 7.72 (dd, J = 8.8, 2.9 Hz, 1 H), 7.32–7.22 (m, 3 H), 7.10–6.99 (m, 3 H), 6.74 (d, J = 3.4 Hz, 1 H), 6.63–6.55 (m, 2 H), 5.74 (s, 1 H), 5.51 (d, J = 15.2 Hz, 1 H), 3.87 (d, J = 15.2 Hz, 1 H), 3.87 (d, J = 15.2 Hz, 1 H), 2.75 (q, J = 7.5 Hz, 2 H), 1.25 (t, J = 7.6 Hz, 3H) (A). 13C (100 MHz, CDCl3) δ163.5, 161.8, 155.7, 148.9, 141.0, 139.3, 132.4, 129.7, 126.3, 122.5, 121.1, 117.3, 116.4, 115.6, 114.6, 67.3, 46.4, 23.5, 15.7. HRMS (ESI): m/z calcd for C21H18F2N2OS [M+H]+: 385.1181, found: 385.1185 (B). (PDF) [file pntd.0005556.s009.pdf]

S2 Text

DHQZ 36 was isolated as an off white/yellow solid (259 mg, 0.75 mmol, 75% yield). NMR ^{1}H (400 MHz, $CDCl_3$) δ 7.72 (dd, *J* = 8.8, 2.9 Hz, 1 H), 7.32-7.22 (m, 3 H), 7.10-6.99 (m, 3 H), 6.74 (d, *J* = 3.4 Hz, 1 H), 6.63-6.55 (m, 2 H), 5.74 (s, 1 H), 5.51 (d, *J* = 15.2 Hz, 1 H), 3.87 (d, *J* = 15.2 Hz, 1 H), 3.87 (d, *J* = 15.2 Hz, 1 H), 2.75 (q, *J* = 7.5 Hz, 2 H), 1.25 (t, *J* = 7.6 Hz, 3H) (A). ^{13}C (100 MHz, $CDCl_3$) δ163.5, 161.8, 155.7, 148.9, 141.0, 139.3, 132.4, 129.7, 126.3, 122.5, 121.1, 117.3, 116.4, 115.6, 114.6, 67.3, 46.4, 23.5, 15.7. HRMS (ESI): m/z calcd for $C_{21}H_{18}F_2N_2OS$ $[M+H]^+$: 385.1181, found: 385.1185 (B).
