# Supplementary material for: Structurally optimized analogs of the retrograde trafficking inhibitor Retro-2cycl limit Leishmania infections
Source: PLoS Negl Trop Dis. 2017 May 15;11(5):e0005556. doi: 10.1371/journal.pntd.0005556 (PMC5444862; doi:10.1371/journal.pntd.0005556)
Supplement: S3 Text — NMR 1H (400 MHz, CDCl3) δ: 7.71 (dd, J = 8.8, 2.9 Hz, 1 H), 7.33–7.24 (m, 3 H), 7.21 (d, J = 3.7 Hz, 1 H), 7.15 (s, 1 H), 7.08–6.98 (m, 3 H), 6.85 (d, J = 3.8 Hz, 1 H), 6.60 (dd, J = 8.7, 4.2 Hz, 1 H), 5.78 (s, 1 H), 5.57 (d, J = 15.3 Hz, 1 H), 3.88 (d, J = 15.3 Hz, 1 H), 2.73 (s, 3 H) (A). 13C (100 MHz, CDCl3) δ 166.7, 163.6, 161.7, 161.1, 158.2, 155.8, 148.5, 141.9, 140.8, 138.8, 132.2, 129.8, 127.0, 123.2, 121.2, 117.3, 116.7, 115.7, 114.6, 112.0, 66.9, 46.5, 19.1. HRMS (ESI): mz/ calcd for C23H17F2N3OS2 [M+H]+: 454.0854, found: 454.0858 (B). (PDF) [file pntd.0005556.s010.pdf]

S3 Text.

DHQZ36.1 was isolated as a yellow solid (203 mg, 0.447 mmol, 89% yield). NMR ^{1}H (400 MHz, $CDCl_3$) δ: 7.71 (dd, *J* = 8.8, 2.9 Hz, 1 H), 7.33-7.24 (m, 3 H), 7.21 (d, *J* = 3.7 Hz, 1 H), 7.15 (s, 1 H), 7.08-6.98 (m, 3 H), 6.85 (d, *J* = 3.8 Hz, 1 H), 6.60 (dd, *J* = 8.7, 4.2 Hz, 1 H), 5.78 (s, 1 H), 5.57 (d, *J* = 15.3 Hz, 1 H), 3.88 (d, *J* = 15.3 Hz, 1 H), 2.73 (s, 3 H) (A). ^{13}C (100 MHz, $CDCl_3$) δ 166.7, 163.6, 161.7, 161.1, 158.2, 155.8, 148.5, 141.9, 140.8, 138.8, 132.2, 129.8, 127.0, 123.2, 121.2, 117.3, 116.7, 115.7, 114.6, 112.0, 66.9, 46.5, 19.1. HRMS (ESI): mz/ calcd for $C_{23}H_{17}F_2N_3OS_2$ $[M+H]^+$: 454.0854, found: 454.0858 (B).
