# Supplementary material for: Structurally optimized analogs of the retrograde trafficking inhibitor Retro-2cycl limit Leishmania infections
Source: PLoS Negl Trop Dis. 2017 May 15;11(5):e0005556. doi: 10.1371/journal.pntd.0005556 (PMC5444862; doi:10.1371/journal.pntd.0005556)
Supplement: S4 Text — Macrophages were then treated with DMSO (vehicle) or DHQZ compounds at concentration ranges of 5–300 μM. For the Max LDH estimation and Spontaneous LDH control, wells were treated with Lysis Buffer or sterile diH2O and incubated for 45 minutes at 37°C for lysis to occur. Plates were spun down and supernatant was collected and measured for LDH at 490 nm and 580 nm background as described in the Pierce LDH Cytotoxicity Assay Kit protocol (ThermoFisher Scientific). DHQZ compound and miltefosine treated cells were compared to the lysis buffer Max LDH control to determine % cytotoxicity. Background was measured and subtracted from wells by subtracting a media control with or without lysis buffer as well as the Spontaneous LDH control. (PDF) [file pntd.0005556.s011.pdf]

LDH Assay. RAW264.7 macrophages were seeded at 2000 cells/well in 96-well plates at 37°C in 5% CO_2 overnight for adherence. Macrophages were then treated with DMSO (vehicle) or DHQZ compounds at concentration ranges of 5-300 µM. For the Max LDH estimation and Spontaneous LDH control, wells were treated with Lysis Buffer or sterile diH_2O and incubated for 45 minutes at 37°C for lysis to occur. Plates were spun down and supernatant was collected and measured for LDH at 490 nm and 580 nm background as described in the Pierce^{TM} LDH Cytotoxicity Assay Kit protocol (ThermoFisher Scientific). DHQZ compound and miltefosine treated cells were compared to the lysis buffer Max LDH control to determine % cytotoxicity. Background was measured and subtracted from wells by subtracting a media control with or without lysis buffer as well as the Spontaneous LDH control.
