# Supplementary material for: Structurally optimized analogs of the retrograde trafficking inhibitor Retro-2cycl limit Leishmania infections
Source: PLoS Negl Trop Dis. 2017 May 15;11(5):e0005556. doi: 10.1371/journal.pntd.0005556 (PMC5444862; doi:10.1371/journal.pntd.0005556)
Supplement: S5 Text — RAW264.7 macrophages were seeded at 3x10^5^ cells/well over glass coverslips in 6-well plates overnight for adherence. Macrophages were then infected with stationary L. amazonensis promastigotes for 24 hours before treatment with Retro-2cycl, DHQZ compounds or miltefosine. For assessment of stimulated macrophages 500 ng/mL LPS and 100 ng/mL IFNγ were added in addition to the drugs and incubated for an additional 24 hours. A replicate set of wells were treated with 10 μM N-acetyl-L-cysteine (NALC) as a superoxide scavenger. Non-infected, infected and infected cells treated with NALC were treated for 24 hours before supernatants were collected and spun down to remove any cellular particles. Supernatants were tested in triplicate for nitrite concentration using the Invitrogen Griess Reagent Kit protocol (ThermoFisher Scientific). (PDF) [file pntd.0005556.s012.pdf]

Griess Reaction. RAW264.7 macrophages were seeded at $3x10^5$ cells/well over glass coverslips in 6-well plates overnight for adherence. Macrophages were then infected with stationary *L. amazonensis* promastigotes for 24 hours before treatment with Retro-2cycl, DHQZ compounds or miltefosine. For assessment of stimulated macrophages 500 ng/mL LPS and 100 ng/mL IFNγ were added in addition to the drugs and incubated for an additional 24 hours. A replicate set of wells were treated with 10 µM N-acetyl-L-cysteine (NALC) as a superoxide scavenger. Non-infected, infected and infected cells treated with NALC were treated for 24 hours before supernatants were collected and spun down to remove any cellular particles. Supernatants were tested in triplicate for nitrite concentration using the Invitrogen Griess Reagent Kit protocol (ThermoFisher Scientific).
