# Supplementary material for: Structurally optimized analogs of the retrograde trafficking inhibitor Retro-2cycl limit Leishmania infections
Source: PLoS Negl Trop Dis. 2017 May 15;11(5):e0005556. doi: 10.1371/journal.pntd.0005556 (PMC5444862; doi:10.1371/journal.pntd.0005556)
Supplement: S6 Text — Promastigotes in early stationary phase were seeded at 5x10^8^ parasites/mL in methionine-free DMEM medium purchased from ThermoFisher and supplemented with 2% dialyzed FBS and 1% penicillin-streptomycin. After 1 hr of methionine starvation, cells were spun down and resuspended in fresh met-free DMEM supplemented with 100μM L-Azidohomoalanine (AHA). Parasites were incubated with AHA supplemented medium for 5 hrs before resuspension in 1% FBS, 1% penicillin-streptomycin Schneider’s medium containing DHQZ analogs or miltefosine at low or high concentrations for 6 hrs. After 6 hrs, cells were spun down and supernatants were collected and treated with 0.1% Triton X-100 for 30 minutes. Aliquots of the supernatant fluid were then frozen at -20°C overnight. They were concentrated by methanol-chloroform precipitation and resuspended in 50mM Tris-HCl, pH 8.0, with 1% SDS as described in the Click-iT Metabolic Labeling Reagents for Proteins protocol for biotinylation (ThermoFisher). Proteins in the suspension were Click-labeled using alkyne biotin purchased from ThermoFisher (Molecular Probes; B10185) and the Click-iT Protein Reaction Buffer Kit (Molecular Probes; C10276). Proteins were then run on Mini-PROTEAN TGX Stain-Free gels purchased from Bio-Rad (Cat: 456–8104) and transferred to nitrocellulose membranes following normal Western blotting procedures. Membranes were blocked in 5% BSA overnight and Click-labeled proteins were detected using a 30 minutes incubation with streptavidin-HRP at a 1:2500 dilution in 2% BSA followed by ECL Western substrate. (PDF) [file pntd.0005556.s013.pdf]

Promastigotes in early stationary phase were seeded at $5x10^8$ parasites/mL in methionine-free DMEM medium purchased from ThermoFisher and supplemented with 2% dialyzed FBS and 1% penicillin-streptomycin. After 1 hr of methionine starvation, cells were spun down and resuspended in fresh met-free DMEM supplemented with 100μM L-Azidohomoalanine (AHA). Parasites were incubated with AHA supplemented medium for 5 hrs before resuspension in 1% FBS, 1% penicillin-streptomycin Schneider's medium containing DHQZ analogs or miltefosine at low or high concentrations for 6 hrs. After 6 hrs, cells were spun down and supernatants were collected and treated with 0.1% Triton X-100 for 30 minutes. Aliquots of the supernatant fluid were then frozen at -20°C overnight. They were concentrated by methanol-chloroform precipitation and resuspended in 50mM Tris-HCl, pH 8.0, with 1% SDS as described in the Click-iT Metabolic Labeling Reagents for Proteins protocol for biotinylation (ThermoFisher). Proteins in the suspension were Click-labeled using alkyne biotin purchased from ThermoFisher (Molecular Probes; B10185) and the Click-iT Protein Reaction Buffer Kit (Molecular Probes; C10276). Proteins were then run on Mini-PROTEAN TGX Stain-Free gels purchased from Bio-Rad (Cat: 456-8104) and transferred to nitrocellulose membranes following normal Western blotting procedures. Membranes were blocked in 5% BSA overnight and Click-labeled proteins were detected using a 30 minutes incubation with streptavidin-HRP at a 1:2500 dilution in 2% BSA followed by ECL Western substrate.
